# Ruthenium-conjugated chrysin analogues modulate platelet activity, thrombus formation and haemostasis with enhanced efficacy

**DOI:** 10.1038/s41598-017-05936-3

**Published:** 2017-07-18

**Authors:** Divyashree Ravishankar, Maryam Salamah, Alda Attina, Radhika Pothi, Thomas M. Vallance, Muhammad Javed, Harry F. Williams, Eman M. S. Alzahrani, Elena Kabova, Rajendran Vaiyapuri, Kenneth Shankland, Jonathan Gibbins, Katja Strohfeldt, Francesca Greco, Helen M. I. Osborn, Sakthivel Vaiyapuri

**Affiliations:** 1 School of Pharmacy, University of Reading, Reading, UK; 2 School of Pharmacy, University of Reading Malaysia, Johar, Malaysia; 3 Institute for Cardiovascular and Metabolic Research, School of Biological Sciences, University of Reading, Reading, UK

## Abstract

The constant increase in cardiovascular disease rate coupled with significant drawbacks of existing therapies emphasise the necessity to improve therapeutic strategies. Natural flavonoids exert innumerable pharmacological effects in humans. Here, we demonstrate the effects of chrysin, a natural flavonoid found largely in honey and passionflower on the modulation of platelet function, haemostasis and thrombosis. Chrysin displayed significant inhibitory effects on isolated platelets, however, its activity was substantially reduced under physiological conditions. In order to increase the efficacy of chrysin, a sulfur derivative (thio-chrysin), and ruthenium-complexes (Ru-chrysin and Ru-thio-chrysin) were synthesised and their effects on the modulation of platelet function were evaluated. Indeed, Ru-thio-chrysin displayed a 4-fold greater inhibition of platelet function and thrombus formation *in vitro* than chrysin under physiologically relevant conditions such as in platelet-rich plasma and whole blood. Notably, Ru-thio-chrysin exhibited similar efficacy to chrysin in the modulation of haemostasis in mice. Increased bioavailability and cell permeability of Ru-thio-chrysin compared to chrysin were found to be the basis for its enhanced activity. Together, these results demonstrate that Ru-thio-coupled natural compounds such as chrysin may serve as promising templates for the development of novel anti-thrombotic agents.

## Introduction

Cardiovascular diseases (CVDs) are collectively regarded as the number one killer worldwide, and notably, thrombosis is responsible for the majority of CVD-associated mortalities and morbidities^1, 2^. Platelets (small circulating blood cells) play indispensable roles in haemostasis by preventing excessive blood loss upon vascular damage through blood clotting. However, inappropriate activation of platelets leads to thrombosis (formation of blood clots within blood vessels) under pathological conditions such as the rupture of atherosclerotic plaques^3^. Thrombosis reduces the blood supply to vital organs such as the heart and brain resulting in heart attacks and strokes, respectively. Hence, targeting platelets has been proven to be effective in the prevention and treatment of CVDs (primarily heart attacks and strokes)^4, 5^. While the currently used anti-platelet drugs such as aspirin and clopidogrel demonstrate efficacy in many patients, they exert undesirable side effects such as bleeding complications and are ineffective in others^5^. Therefore, the development of safer, more effective therapeutic strategies for the prevention and treatment of thrombotic diseases is a pressing priority.

The direct relationships between dietary components and cardiovascular health have been established over the last few decades even though their underlying molecular mechanisms are not well understood^6–9^. Although several genetic factors account for the development of CVD risks in multiple settings^10^, dietary components form an essential part of the disease progression. While a number of dietary molecules such as lipids are responsible for the development of CVDs, many small molecules including flavonoids that are present in various plant products exert beneficial effects in the prevention of such diseases^11, 12^. Individuals who consume diets with low levels of saturated fatty acids together with a substantial amount of fruits and vegetables have been shown to have reduced development of CVD risks^13, 14^. In particular, a number of dietary flavonoids such as quercetin^15^, tangeretin^16^, nobiletin^17^, luteolin and apigenin have been shown to exhibit inhibitory effects in platelets by modulating diverse signalling cascades^18^. However, several challenges are associated with using dietary components for the prevention and treatment of diseases. Some of these include malabsorption, poor bioavailability of desired compounds in the blood stream, poor metabolic stability as well as reduced lipophilicity to readily cross the cell membranes^19–22^. Specific chemical modifications (e.g. addition of sulfur groups^23^) to the fundamental structures of dietary flavonoids have been shown to improve their hydrophobic nature and enhance their biological activities. Indeed, all the natural flavonoids share a basic template structure which can be modified by the specific addition of various functional groups, affording a diverse array of biological activities^18, 22^.

In recent years, the therapeutic applications of organometallic complexes have been considered for numerous pathological conditions^24–27^. For example, cisplatin, a platinum-based FDA-approved anti-cancer drug has substantially increased the survival rate of patients with testicular cancer^28^ and it is being widely used to treat different types of cancer^29^. The success of cisplatin, as well as the occurrence of dose-limiting side effects stimulated significant research in this area. Ruthenium-based organometallics are a very promising class of therapeutic compound, with two specific candidates, NAMI-A and KP1019 having entered clinical trials^30, 31^. There are three specific properties which make ruthenium an interesting metal for drug development: its range of oxidation states, its ability to mimic iron binding under physiological conditions and its low toxicity compared to platinum^32^. Hence, we hypothesised that ruthenium-based complexes of flavonoids may offer greater efficacy in target cell types by overcoming the problems associated with the natural flavonoids. In this study, we report the design, synthesis, chemical characterisation and biological evaluation of novel ruthenium complexes of chrysin, a natural flavonoid present in honey, honeycombs, propolis and passionflowers, as well as its synthesised thioflavone derivative for the modulation of platelet function, *in vitro* thrombus formation and haemostasis.

## Results

### Synthesis and chemical characterisation of the ruthenium complexes of chrysin and thio-chrysin

**Figure 1:**
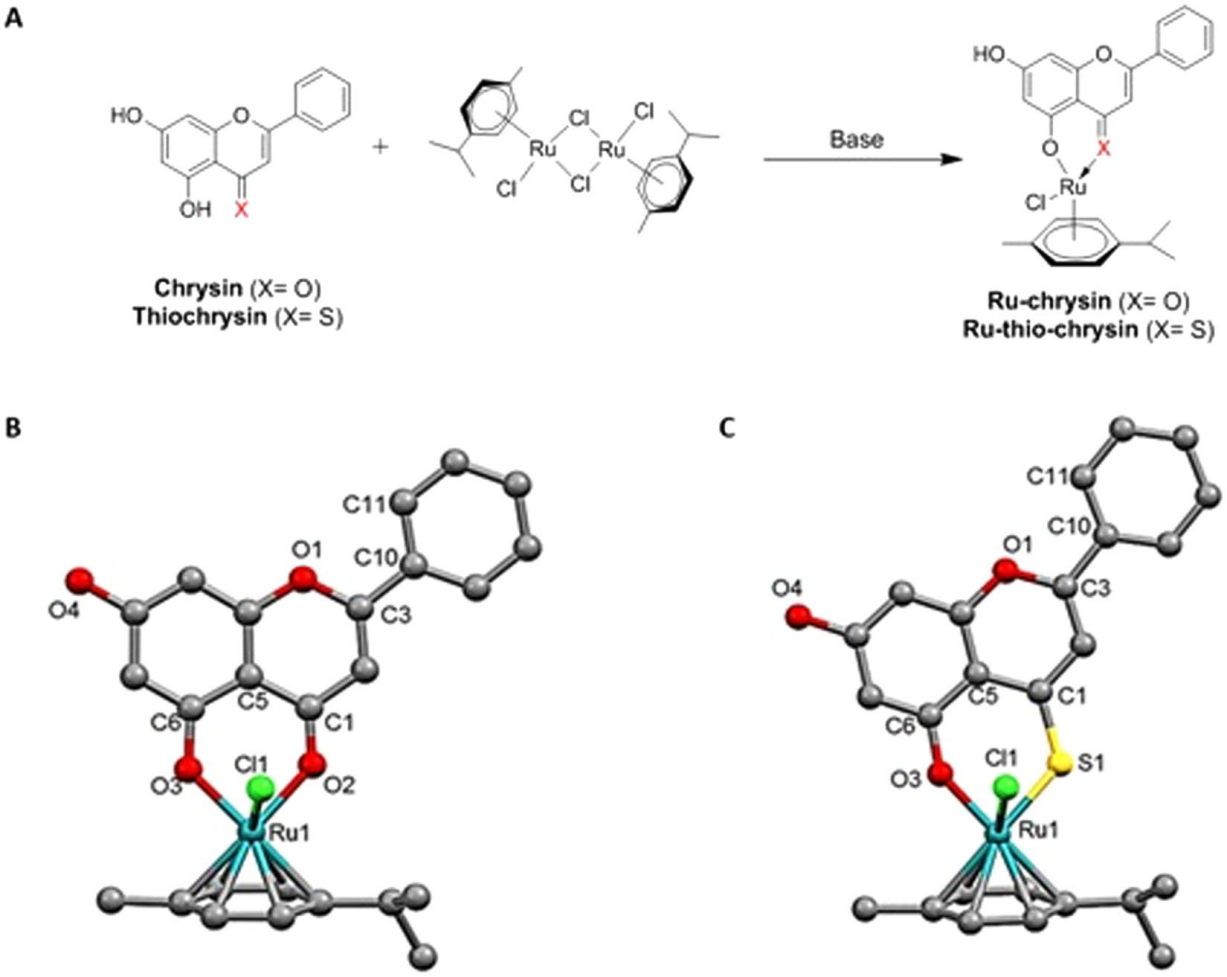
Synthesis scheme and molecular structures for Ru-chrysin and Ru-thio-chrysin. (**A**) Schematic diagram represents the synthesis of Ru-chrysin and Ru-thio-chrysin from chrysin and thio-chrysin, respectively *via* reaction with bis [dichlorido (η^6^-*p*-cymene)] ruthenium(II). The molecular structures of Ru-chrysin (**B**) and Ru-thio-chrysin (**C**) were determined by X-ray diffraction studies. The hydrogen bonds are not shown in the figures to enhance the clarity of the molecular images. The symbols C, O, Cl, S and Ru represent carbon, oxygen, chlorine, sulfur and ruthenium, respectively. The numbers shown indicate the position of respective carbon atoms.

Similar to a recent study^33^, our initial experiments confirmed the inhibitory effects of chrysin in the activation of washed platelets in a concentration-dependent manner. Due to the limited knowledge of chrysin in the modulation of platelet function, thrombosis and haemostasis, this flavonoid was selected as a starting point for the chemical synthesis and biological evaluation of derivatives with additional chemical features in order to achieve enhanced platelet inhibitory effects under physiological conditions. In order to determine the relative potency of flavones versus thioflavones, thio-chrysin was chemically synthesised as reported by us previously^23^. New methods were then developed in order to access the novel ruthenium-chrysin (Ru-chrysin) and ruthenium-thio-chrysin (Ru-thio-chrysin) complexes. Chrysin and thio-chrysin were initially deprotonated prior to reacting with the commercially available bis [dichlorido (η^6^-*p*-cymene)] ruthenium(II), as shown in the scheme (Fig. 1A). Optimisation of synthesis was performed to ensure efficient deprotonation of the phenolic hydroxyl group adjacent to the carbonyl residue prior to the addition of the organometallic reagents. Table S1 displays the range of conditions used with different bases and molar equivalents (eq.) of NaOMe (1.05 eq. to 2.00 eq.) to synthesise Ru-chrysin. Product isolation proved to be difficult in the presence of Hünig’s base, triethyl amine, or DBU. Reactions with 1.10 eq., 1.25 eq. and 2.00 eq. of NaOMe yielded a mixture of products with the starting material (chrysin) being the primary contaminant. Purification of Ru-chrysin from these mixtures through recrystallisation was unsuccessful. ^1^H NMR spectroscopic analysis of the reaction mixtures revealed a positive correlation between the percentage of uncomplexed chrysin and an increased molar equivalent of NaOMe. This indicated that the excess amount of NaOMe might be affecting the hydrolysis of the bis [dichlorido(η^6^-*p*-cymene)] ruthenium(II) reagent thus leading to a higher percentage of uncomplexed chrysin. Finally, complete conversion of chrysin to Ru-chrysin was achieved in the presence of 1.05 eq. of NaOMe with 0.55 eq. of bis [dichlorido(η^6^-*p*-cymene)] ruthenium(II). The product Ru-chrysin was purified by recrystallisation from EtOAc:CHCl_3_ (9:1 v/v) and was isolated in a yield of 75%.

The conditions used for preparing Ru-chrysin (1.05 eq. of NaOMe with 0.55 eq. of bis [dichlorido(η^6^-*p*-cymene)] ruthenium(II)) yielded the Ru-thio-chrysin product but with a higher percentage of uncomplexed starting material, thio-chrysin. As illustrated above, an increased quantity of NaOMe was not beneficial in achieving a complete reaction conversion and therefore the synthesis of Ru-thio-chrysin was attempted with 0.90 eq. of bis [dichlorido (η^6^-*p*-cymene)] ruthenium(II) and 1.05 eq. of NaOMe. This reaction successfully yielded Ru-thio-chrysin and purification was achieved by recrystallisation from EtOAc:CHCl_3_ (9:1 v/v) which resulted in access to the pure material in 65% yield. The loss of one proton in the ^1^H NMR spectra of Ru-chrysin (Figure S1A) *versus* chrysin, and Ru-thio-chrysin (Figure S2) *versus* thio-chrysin, from the hydroxyl groups of chrysin and thio-chrysin, respectively, confirmed the formation of Ru-complexes. A range of further chemical characterisation methods including ^13^C-NMR spectroscopic analysis (Figure S1B), mass spectrometry and elemental analysis confirmed the formation of the Ru-complexes. Furthermore, the crystal structures of Ru-chrysin (Fig. 1B) and Ru-thio-chrysin (Fig. 1C) were solved from powder X-ray diffraction data (Figure S3, Table S2) using DASH^34, 35^ and refined using TOPAS (Bruker, Germany), as only polycrystalline samples of both compounds could be obtained.

### Ru-chrysin and Ru-thio-chrysin display enhanced effects in platelet-rich plasma compared to washed platelets

**Figure 2:**
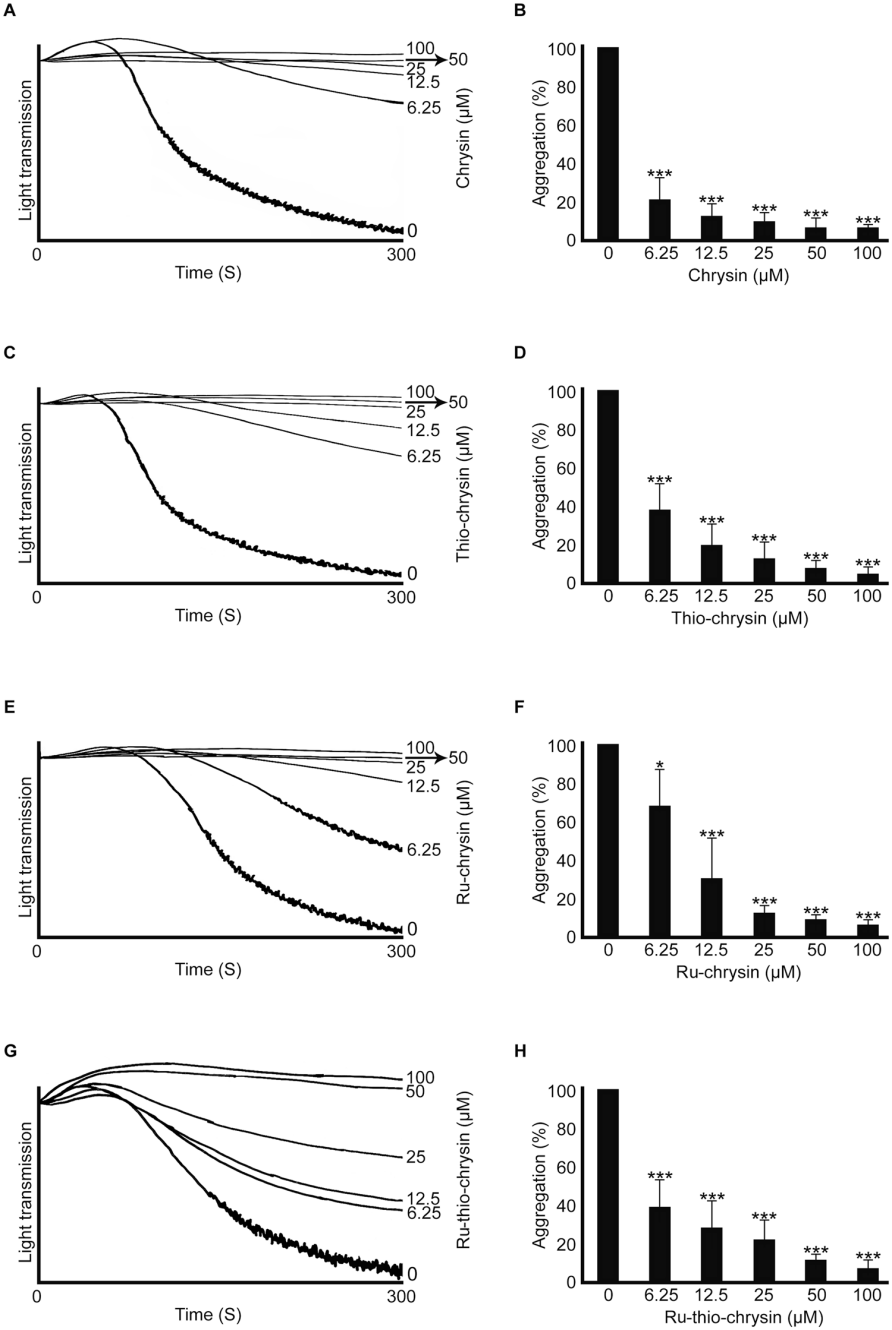
Effects of chrysin and its derivatives in washed platelet aggregation. Human washed platelets were incubated with a vehicle control [0.1% (v/v) DMSO] or different concentrations of chrysin (**A**,**B**), thio-chrysin (**C**,**D**), Ru-chrysin (**E**,**F**) and Ru-thio-chrysin (**G**,**H**) for 5 minutes prior to the addition of 0.5 μg/mL CRP-XL and the platelet aggregation was monitored for 5 minutes by optical aggregometry. The aggregation traces shown are representative of three separate experiments. The maximum aggregation obtained with vehicle control at 5 minutes was taken as 100% to calculate the level of aggregation for chrysin and its derivatives-treated samples. Cumulative data represent mean ± S.D. (n = 3). *Indicates significance with respect to controls and *p* values shown (*p < 0.05, and ***p < 0.001) are as calculated by one-way ANOVA using Graphpad Prism.

To determine the effects of chrysin and thio-chrysin and their Ru-complexes in the modulation of platelet activation, aggregation assays were performed using cross-linked collagen-related peptide (CRP-XL) as a platelet agonist, by optical aggregometry. Firstly, to determine the effects of chrysin and its derivatives on isolated platelets, aggregation assays were performed using human washed platelets (devoid of plasma proteins and other blood cells). Chrysin inhibited CRP-XL (0.5 μg/mL)-induced aggregation in washed platelets in a concentration-dependent manner (Fig. 2A and B). A concentration of 6.25 μM chrysin displayed approximately 80% reduction in CRP-XL (0.5 μg/mL)-induced platelet aggregation. The addition of a thiol group to chrysin did not affect its inhibitory effects (Fig. 2C and D). While Ru-Cl failed to affect platelet aggregation in washed platelets (Figure S4A), Ru-chrysin (Fig. 2E and F) and Ru-thio-chrysin (Fig. 2G and H) inhibited CRP-XL-induced platelet aggregation in a concentration-dependent manner. The overall effects of Ru-thio-chrysin were significantly better than Ru-chrysin at least at lower concentrations such as 6.25 μM, although higher concentrations resulted in similar effects. Together, these data demonstrate that the chemical modifications of chrysin did not affect its overall inhibitory activities in human washed platelets.

**Figure 3:**
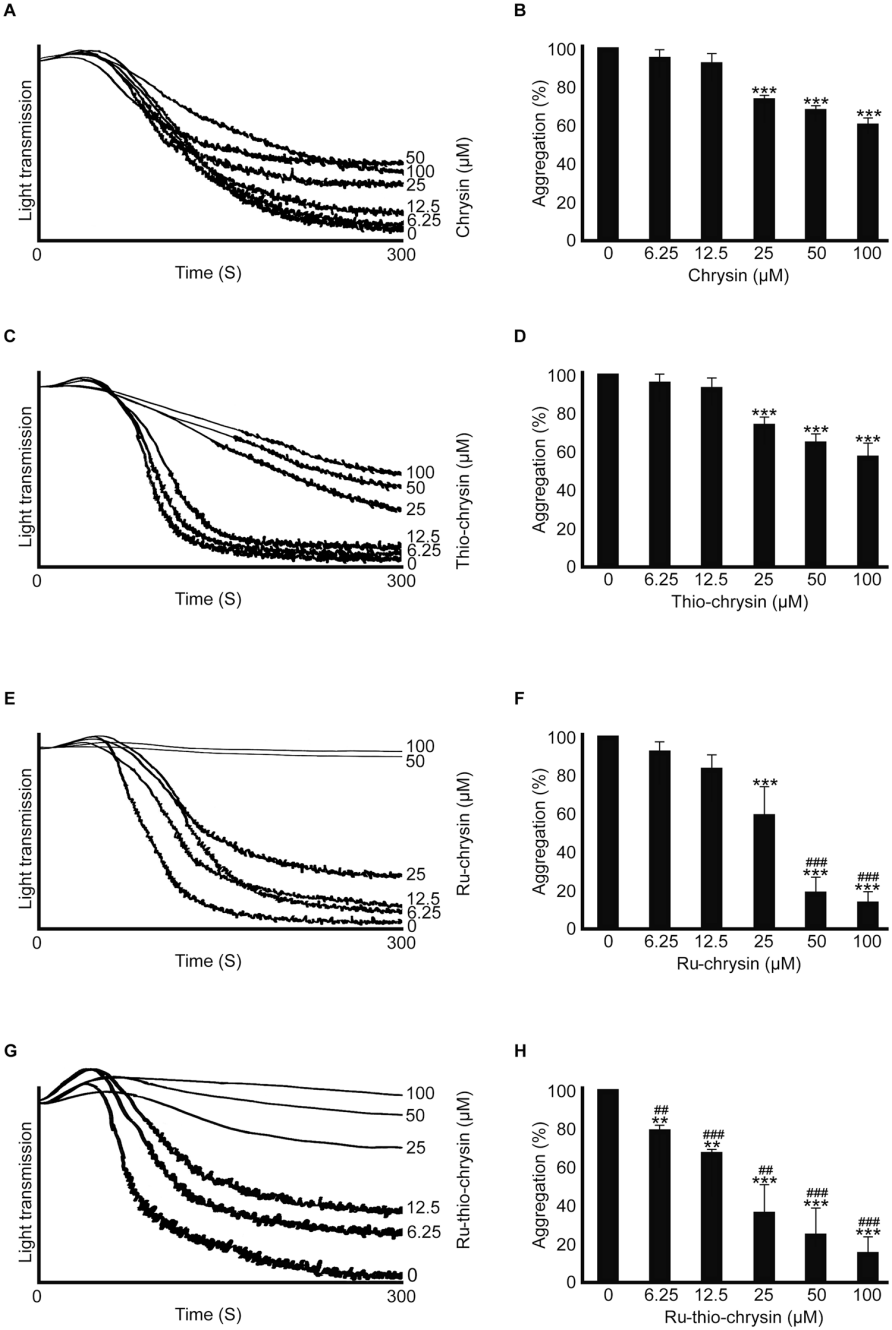
Impact of chrysin and its derivatives on platelet activation in platelet-rich plasma. Human platelet-rich plasma was treated with vehicle control [0.1% (v/v) DMSO] or a range of concentrations of chrysin (**A**,**B**), thio-chrysin (**C**,**D**), Ru-chrysin (**E**,**F**) and Ru-thio-chrysin (**G**,**H**) for 5 minutes prior to the addition of 0.5 μg/mL CRP-XL in an optical aggregometer. The platelet aggregation was monitored for 5 minutes. The traces shown are representative of three separate experiments. The maximum aggregation obtained with vehicle control at 5 minutes was taken as 100% to calculate the level of aggregation for chrysin and its derivatives-treated samples. Cumulative data represent mean ± S.D. (n = 3). *Indicates significance with respect to controls and ^#^indicates significance with respect to the respective chrysin concentrations, *p* values shown (**^,##^ *p* < 0.01 and ***^,###^ *p* < 0.001) are as calculated by one-way ANOVA using Graphpad Prism.

Plasma proteins such as albumin have been shown to bind small molecules including flavonoids and affect their bioavailability to target cells^36^. Therefore, to determine the effects of various concentrations of chrysin and its derivatives in the presence of plasma proteins, aggregation assays were performed using human platelet-rich plasma (PRP) and 0.5 μg/mL CRP-XL. In contrast to the results obtained with washed platelets, chrysin displayed reduced inhibitory effects on platelet aggregation in PRP. A concentration of 100 μM chrysin displayed around 40% reduction in platelet aggregation upon activation with 0.5 μg/mL CRP-XL, whilst the lower concentrations such as 6.25 μM and 12.5 μM did not exhibit significant effects (Fig. 3A and B). Thio-chrysin displayed similar inhibitory effects compared to chrysin in platelet aggregation (Fig. 3C and D). As with washed platelets, Ru-Cl failed to show any inhibitory effects in PRP (Figure S4B). While Ru-chrysin did not significantly affect the platelet aggregation at the concentrations of 6.25 μM and 12.5 μM (Fig. 3E and F), Ru-thio-chrysin displayed a significant effect at lower concentrations including 6.25 μM compared to the control (Fig. 3G and H). In addition, Ru-thio-chrysin exhibited significantly more inhibitory effects compared to the respective concentrations of native chrysin. These data demonstrate that although chrysin exhibited enhanced inhibitory effects on platelet activation in washed platelets, it failed to show similar levels of effects in PRP. However, Ru-chrysin and Ru-thio-chrysin displayed enhanced inhibitory effects on platelets even in PRP. The Ru-complex of thio-chrysin appeared to be more effective than others in the modulation of platelet activation in the presence of plasma proteins.

### Chrysin and its derivatives affect inside-out signalling to integrin αIIbβ3

**Figure 4:**
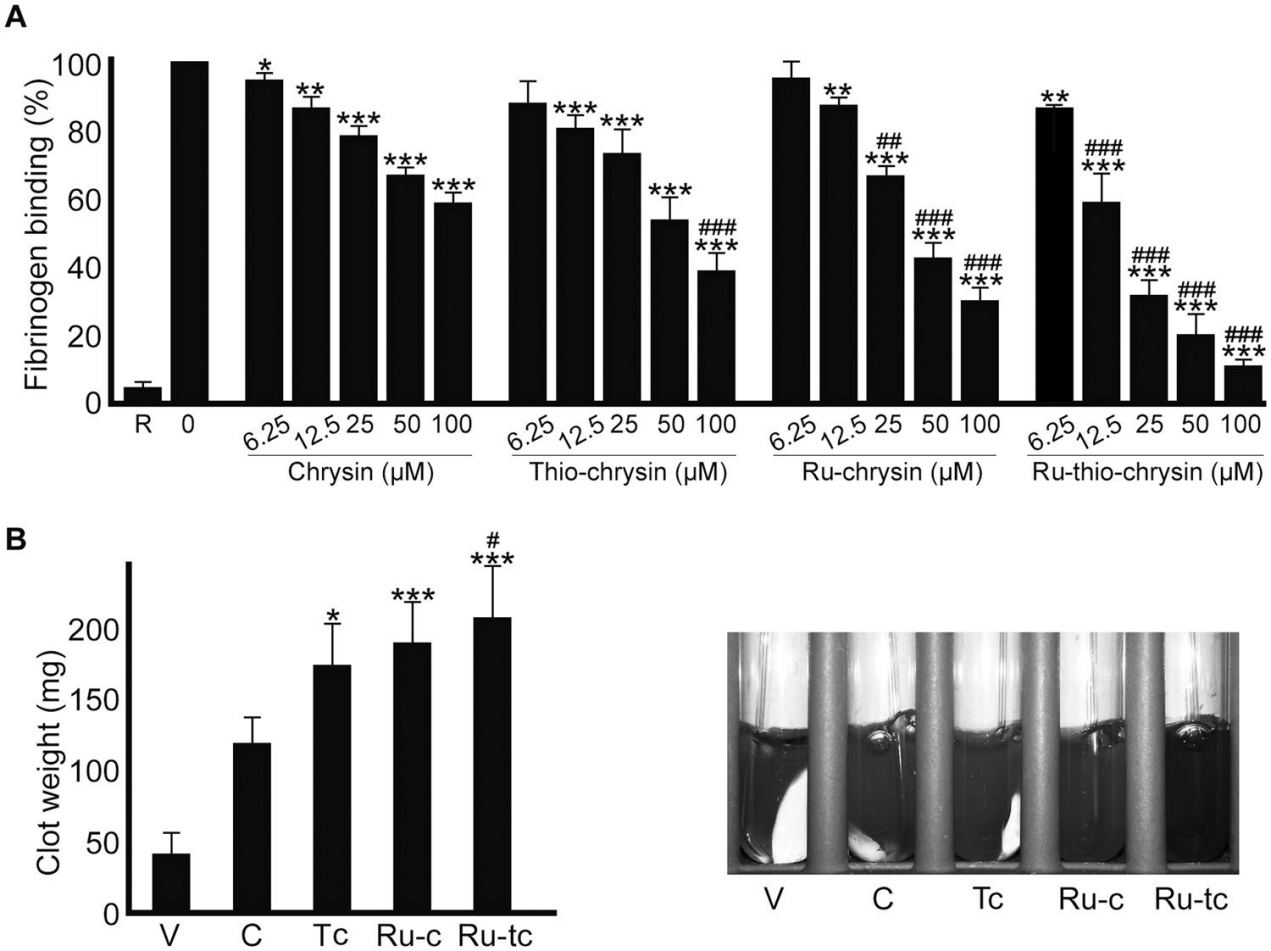
Chrysin and its derivatives inhibit inside-out and outside-in signalling in platelets. (**A**) human platelet-rich plasma was treated with vehicle [0.1% (v/v) DMSO] or diverse concentrations of chrysin, thio-chrysin, Ru-chrysin and Ru-thio-chrysin for 5 minutes prior to the addition of 0.5 μg/mL CRP-XL and incubation of 20 minutes at room temperature. The level of fibrinogen binding (as a marker for inside-out signalling to integrin αIIbβ3) was quantified using FITC-labelled anti-human fibrinogen antibodies by flow cytometry. The level of fluorescence obtained with vehicle control was taken as 100% to calculate the extent of inhibition in chrysin and its derivatives-treated samples. R represents ‘resting’ platelets. Cumulative data represent mean ± S.D. (n = 4). (**B**) human platelet-rich plasma was treated with vehicle (V) [0.1% (v/v) DMSO] or 50 μM of chrysin (C) or its derivatives, thio-chrysin (Tc), Ru-chrysin (Ru-c) and Ru-thio-chrysin (Ru-tc) for 5 minutes. Following incubation, clotting was initiated by the addition of 1 U/mL thrombin and the clot retraction was monitored for three hours. The remaining clot weight at three hours was measured to analyse the extent of retraction process. The image shown on the right is representative of four separate experiments. *Indicates significance with respect to controls and ^#^indicates significance with respect to the respective chrysin concentrations; *p* values shown (*^,#^ *p* < 0.05, **^,##^ *p* < 0.01 and ***^,###^ *p* < 0.001) are as calculated by one-way ANOVA using Graphpad Prism.

Since chrysin and its derivatives affected platelet aggregation, we hypothesised that they may affect inside-out signalling to integrin αIIbβ3, as this plays a critical role in the affinity modulation of this integrin and its subsequent binding to fibrinogen in order to facilitate platelet aggregation^3^. Hence, the level of fibrinogen binding on the platelet surface was measured as a marker for inside-out signalling to integrin αIIbβ3 using human PRP and FITC-labelled anti-fibrinogen antibodies by flow cytometry. Similar to aggregation assays, Ru-thio-chrysin showed significantly enhanced inhibitory effects on fibrinogen binding to the platelet surface upon activation with 0.5 μg/mL CRP-XL (Fig. 4A). While the concentration of 6.25 μM Ru-thio-chrysin showed around 15–20% inhibition, it inhibited the fibrinogen binding levels by almost 90% at 100 μM concentration. Notably, Ru-thio-chrysin (at concentrations of more than 12.5 μM) and Ru-chrysin (at concentrations of more than 25 μM) displayed significantly more effects compared to the respective concentrations of chrysin. Chrysin and thio-chrysin displayed similar inhibitory effects. Similar to the aggregation data, these results further emphasise the increased potential of Ru-complexes in exerting their target effects under these conditions.

### Outside-in signalling driven by integrin αIIbβ3 is affected by chrysin and its derivatives

Following fibrinogen binding, integrin αIIbβ3 triggers signalling into the platelets in order to induce clot retraction and promote wound healing. This also represents a late downstream signalling event in the platelet activation process^37^. To determine whether chrysin and its derivatives affect outside-in signalling by integrin αIIbβ3, a clot retraction assay was performed. The human platelets (PRP) were treated with different concentrations of chrysin, thio-chrysin, Ru-chrysin and Ru-thio-chrysin prior to the addition of thrombin and initiation of clot formation and subsequent retraction. At three hours, the clot obtained with the vehicle control [0.1% (v/v) DMSO] retracted to around 50 mg. Although chrysin did not show significant effects, its derivatives substantially inhibited the clot retraction process as the clot weights remained at more than 100 mg (Fig. 4B). Similar to other platelet functional assays, Ru-thio-chrysin displayed superior effects in comparison to native chrysin. Interestingly, thio-chrysin displayed significant inhibitory effect in clot retraction, suggesting that the chemical modification of 4-C=O (carbonyl) to 4-C=S (thiocarbonyl) may influence integrin αIIbβ3-mediated outside-in signalling. Together, these data establish a role for chrysin derivatives in the modulation of outside-in signalling by integrin αIIbβ3 in human platelets.

### Effects of chrysin and its derivatives on granule secretion in platelets

**Figure 5:**
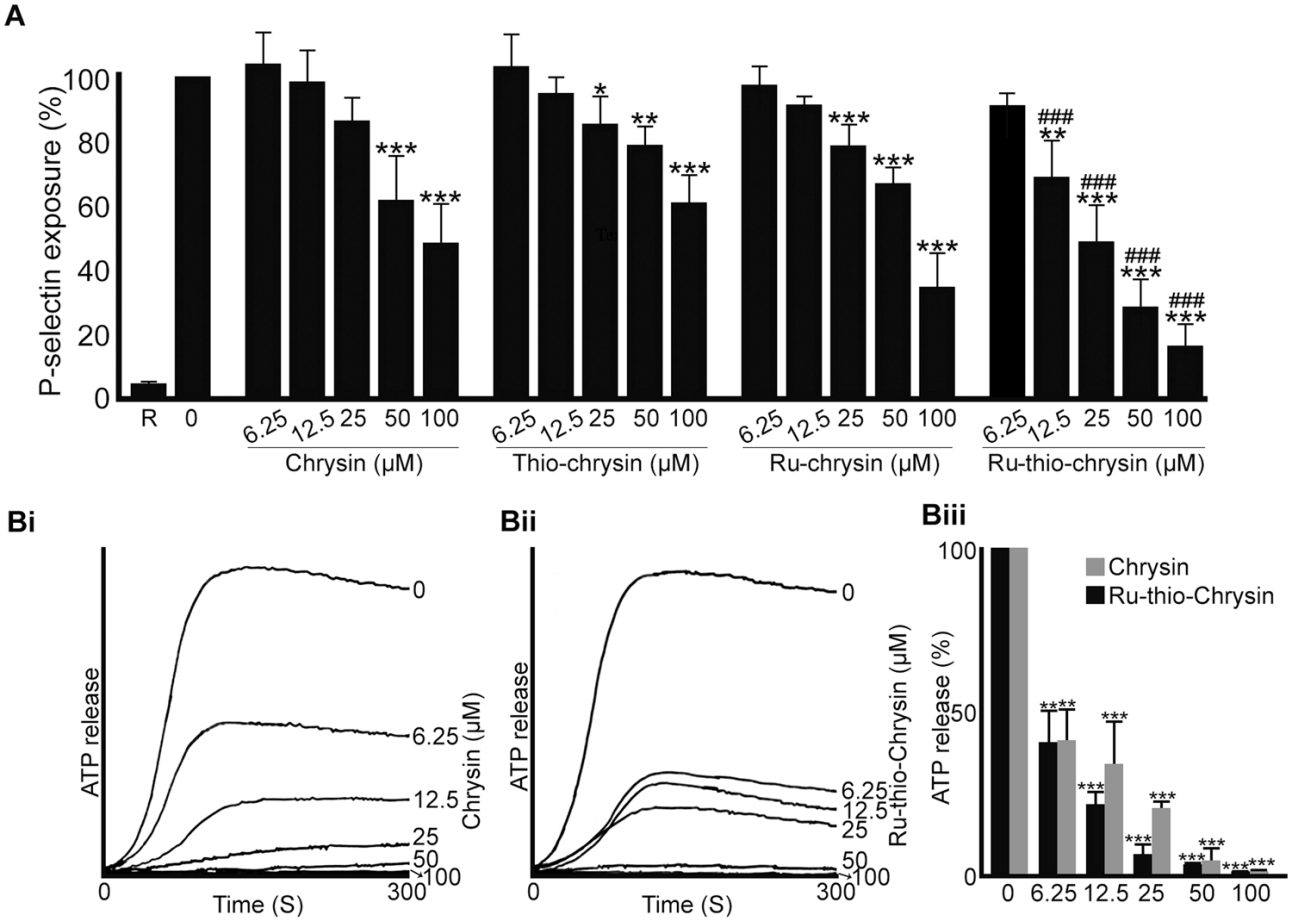
Chrysin and its derivatives affect granule secretion in platelets. (**A**) human platelet-rich plasma was treated with vehicle [0.1% (v/v) DMSO] or diverse concentrations of chrysin, thio-chrysin, Ru-chrysin and Ru-thio-chrysin for 5 minutes prior to the addition of 0.5 μg/mL CRP-XL and incubation of 20 minutes at room temperature. The level of P-selectin (as a marker for α-granule secretion) was quantified using PECy5-labelled anti-human P-selectin antibodies by flow cytometry. The level of fluorescence obtained with vehicle control was taken as 100% to calculate the extent of inhibition in chrysin and its derivatives-treated samples. R represents ‘resting’ platelets. Cumulative data represent mean ± S.D. (n = 4). (**B**) human washed platelets were mixed with luciferin-luciferase reagent for two minutes followed by incubation with a vehicle control [0.1% (v/v) DMSO] or different concentrations of chrysin (**i**) or Ru-thio-chrysin (**ii**) for another 5 minutes. Platelets were then activated with 0.5 μg/mL CRP-XL and the ATP release was monitored for 5 minutes by lumi-aggregometry. The traces shown are representative of three separate experiments. The maximum ATP release obtained with vehicle control was taken as 100% to calculate the level of inhibition in chrysin and Ru-thio-chrysin treated samples (**iii**). Cumulative data represent mean ± S.D. (n = 3). *Indicates significance with respect to controls and ^#^indicates significance with respect to the respective chrysin concentrations; *p* values shown (*p < 0.05, **p < 0.01 and ***^,###^ *p* < 0.001) are as calculated by one-way ANOVA using Graphpad Prism.

Platelets primarily contain two types of granules (α- and dense granules) and the activation of platelets releases the granule contents to the external environment, where they play important roles in the stimulation of additional platelets and their recruitment to the growing thrombus^38^. To establish whether chrysin and its derivatives affect α-granule secretion in platelets, the level of P-selectin on the platelet surface upon activation with 0.5 μg/mL CRP-XL was measured as a marker for α-granule secretion using human PRP and PECy5-labelled anti-P-selectin antibodies by flow cytometry. CRP-XL-induced α-granule secretion was inhibited significantly by Ru-thio-chrysin at the concentrations of 12.5 μM and above, with approximately 80% inhibition being achieved at 100 μM concentration (Fig. 5A). Ru-chrysin and thio-chrysin significantly affected platelet granule secretion at the concentrations of 25 μM and above. However, chrysin affected α-granule secretion only at a minimum concentration of 50 μM in platelets. Only Ru-thio-chrysin displayed more inhibitory effects on α-granule secretion compared to chrysin at concentrations from 12.5 μM in PRP.

Based on the above data, chrysin and Ru-thio-chrysin were selected to distinguish their modulatory effects on dense granule secretion and other platelet functions. ATP secretion was measured as a marker for dense granule secretion in washed platelets upon activation with 0.5 μg/mL CRP-XL in the presence and absence of different concentrations of chrysin and Ru-thio-chrysin using a luciferin-luciferase luminescence assay (Fig. 5B). Both chrysin and Ru-thio-chrysin significantly inhibited dense granule secretion at the concentrations tested (6.25–100 μM) with 100% inhibition observed at 100 μM concentration of both. These data demonstrate that chrysin and its derivatives significantly affect platelet granule secretion, which may influence the subsequent functions of platelets.

### Chrysin and Ru-thio-chrysin do not affect platelet adhesion under static conditions

Integrins αIIbβ3 and α2β1 play critical roles in platelet adhesion to different matrix proteins such as fibrinogen and collagen, respectively. To determine whether chrysin and Ru-thio-chrysin directly influence platelet adhesion by affecting the functions of integrins αIIbβ3 and α2β1, the static platelet adhesion assay was performed on collagen, CRP-XL (a selective ligand for GPVI that is used to differentiate the binding effects of collagen to GPVI and integrin α2β1) and fibrinogen-coated surfaces using human PRP in the presence and absence of various concentrations of chrysin and Ru-thio-chrysin. Moreover, to determine the linear impact of these compounds on integrin αIIbβ3, integrilin (4 µM), an antagonist for integrin αIIbβ3 was also used in this assay prior to the treatment with different concentrations of chrysin and Ru-thio-chrysin. Chrysin and Ru-thio-chrysin did not affect platelet adhesion to collagen, CRP-XL or fibrinogen both in the presence and absence of integrilin (Figure S5). These data suggest that chrysin and Ru-thio-chrysin may not directly affect the functions of αIIbβ3 and α2β1 on the platelet surface.

### Effects of chrysin and Ru-thio-chrysin in the modulation of calcium signalling in platelets

**Figure 6:**
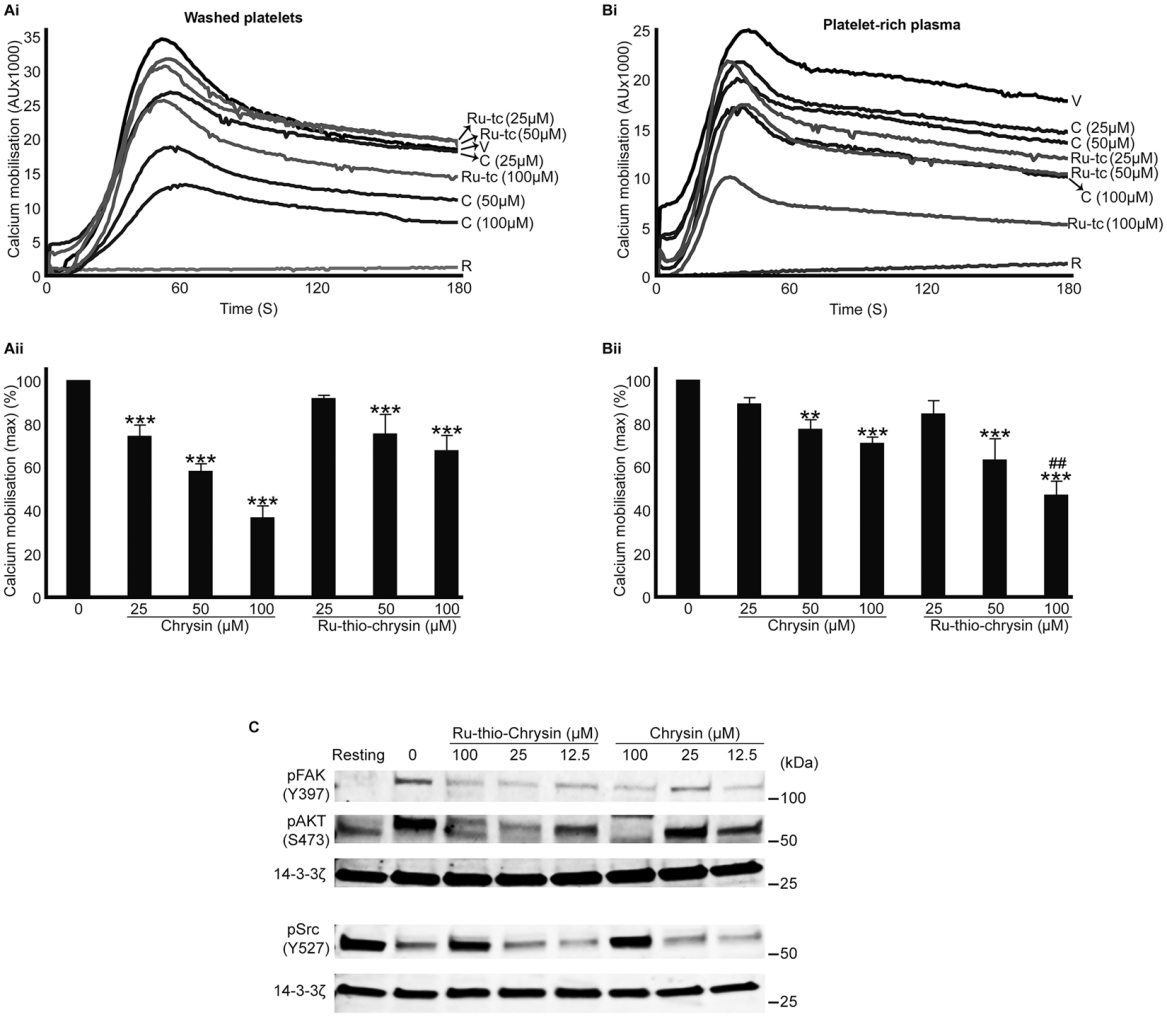
Chrysin and its derivatives influence the calcium mobilisation and phosphorylation of various signalling proteins in platelets. Fluo4 AM-dye labelled human washed platelets (**Ai**,**Aii**) and platelet-rich plasma (**Bi**,**Bii**) were treated with vehicle control (V) [0.1% (v/v) DMSO] or different concentrations of chrysin (C) or Ru-thio-chrysin (Ru-tc) for 5 minutes prior to the addition of 0.5 μg/mL CRP-XL and monitoring of calcium mobilisation for three minutes by flourimetry. The calcium traces (**Ai** and **Bi**) shown are representative of three separate experiments. The maximum fluorescence obtained with each sample was converted into percentages to calculate the level of calcium mobilisation obtained with vehicle and chrysin or Ru-thio-chrysin treated samples. Cumulative data (**Aii** and **Bii**) represent mean ± S.D. (n = 3). *Indicates significance with respect to controls and ^#^indicates significance with respect to the respective chrysin concentrations; *p* values shown (**^,##^ *p* < 0.01 and ***p < 0.001) are as calculated by one-way ANOVA using Graphpad Prism. (**C**) Human washed platelets were treated with vehicle control or different concentrations of chrysin or Ru-thio-chrysin for 5 minutes prior to the addition of 0.5 μg/mL CRP-XL and incubation for another 5 minutes. The platelets were lysed and used for immunoblot analysis to detect the level of AKT phosphorylation at residue S473, FAK phosphorylation at Y397 and phosphorylation of Src at Y527. The level of 14-3-3ζ protein was detected as a loading control. The cropped images of the blots shown here are representative of three separate experiments. The uncropped full length blots are presented in Supplementary Information (Figure S8).

Platelets contain a dense-tubular system to store calcium, and the activation of platelets allows the mobilisation of calcium from stores to the cytoplasm. Similarly, a substantial amount of calcium is also pumped from the external milieu into the cytoplasm during platelet activation. The elevated levels of calcium play major roles including the intracellular reorganisation of cytoskeleton to allow platelet spreading and subsequent thrombus formation^39^. Based on the above results achieved in washed platelets and PRP, chrysin and Ru-thio-chrysin were tested to evaluate their effects on the modulation of calcium mobilisation. Chrysin displayed greater inhibitory effects on calcium mobilisation than Ru-thio-chrysin in washed platelets compared to the control (Fig. 6Ai and Aii). However, Ru-thio-chrysin exhibited significantly greater effects in PRP compared to chrysin at a concentration of 100 μM (Fig. 6Bi and Bii). Similar to other assays performed using PRP, Ru-thio-chrysin showed enhanced reduction in calcium mobilisation in platelets with around 60% obtained at 100 μM. Together, these results demonstrate the impact of chrysin and Ru-thio-chrysin in the modulation of calcium mobilisation in platelets.

### Chrysin and Ru-thio-chrysin negatively regulate PI3K/AKT and Src signalling in platelets

Calcium mobilisation is associated with phosphoinositide 3-kinase (PI3K) signalling in platelets^39^. Protein kinase B (AKT) is a downstream effector of PI3K and a key marker for the PI3K/AKT signalling pathway. A recent study has demonstrated the inhibitory effects of chrysin on the phosphorylation of AKT^33^. In addition, chrysin has been reported to inhibit focal adhesion kinase (FAK) activation^33^, which plays a key role in downstream signalling of integrins (outside-in signalling) that leads to platelet spreading^40^ as well as Src kinases that are critical initiators of integrin signalling and platelet activation^41^. Therefore, to determine whether chrysin and Ru-thio-chrysin share similar molecular targets in platelets, the phosphorylation of AKT at S473, FAK at Y397 and Src at Y527 (inhibitory site) was measured using resting and 0.5 μg/mL CRP-XL activated platelets in the presence and absence of different concentrations of chrysin and Ru-thio-chrysin by immunoblot analysis. The vehicle [0.1% (v/v) DMSO]-treated samples displayed a notable level of phosphorylation of AKT and FAK upon activation with CRP-XL, however, chrysin and Ru-thio-chrysin inhibited their phosphorylation in a concentration-dependent manner (Fig. 6C). Similarly, chrysin and Ru-thio-chrysin had a significant impact on the dephosphorylation of Src at Y527, which is an essential phenomenon for the activation of platelets. Together these data suggest that chrysin and Ru-thio-chrysin may share similar molecular targets in platelets, and their inhibitory effects on Src may directly or indirectly influence other signalling pathways that render the inhibition of platelet function.

### Chrysin and Ru-thio-chrysin inhibit thrombus formation under arterial flow conditions

**Figure 7:**
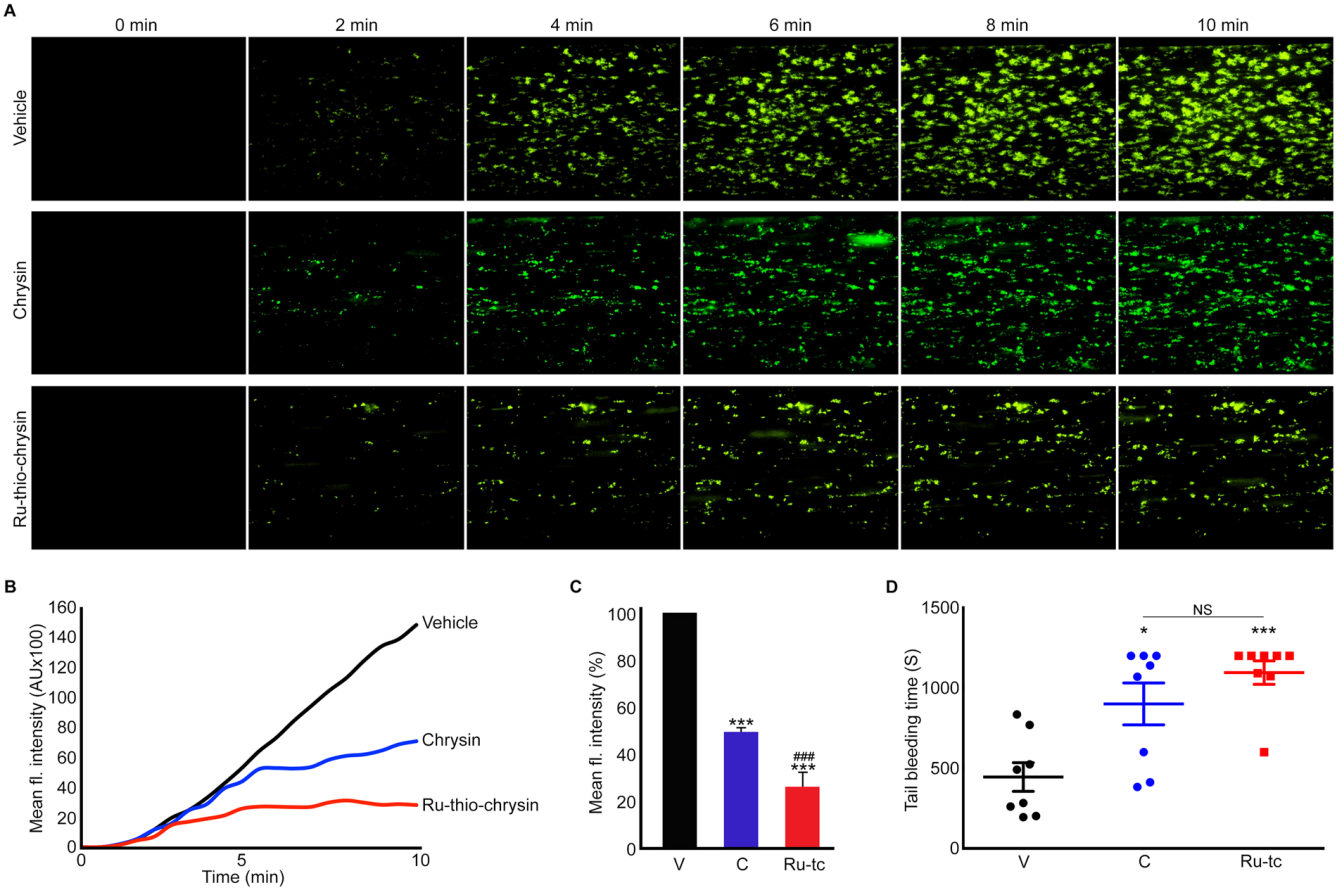
Differential effects of chrysin and Ru-thio-chrysin in the modulation of thrombus formation under arterial flow conditions and haemostasis in mice. DiOC6-labelled human whole blood was treated with vehicle control [0.1% (v/v) DMSO] or 100 μM chrysin or Ru-thio-chrysin for 5 minutes prior to the perfusion over collagen-coated Vena8 biochips. Thrombus formation was monitored by capturing Z-stack fluorescent images at every 30 seconds over 10 minutes by fluorescence microscopy. The images shown (**A**) at different time points and traces (**B**) are representative of three separate experiments. (**C**) The mean fluorescence intensity obtained with vehicle control (V) at 10 minutes was taken as 100% to calculate the level of inhibition in chrysin (C) and Ru-thio-chrysin (Ru-tc)-treated samples. Data represent mean ± S.D. (n = 3). *Indicates significance with respect to control and ^#^indicates significance with respect to the respective chrysin concentration; *p* values shown (***^,###^ *p* < 0.001) are as calculated by one-way ANOVA using Graphpad Prism. (**D**) The effects of vehicle control [0.1% (v/v) DMSO] or 25 μM of chrysin (C) or Ru-thio-chrysin (Ru-tc) in the modulation of haemostasis were analysed by tail bleeding assay in mice. Data represent ± S.D. (n = 8 mice in each group). The *p* values shown (*p < 0.05 and ***p < 0.001) are as calculated by non-parametric Mann-Whitney test using Graphpad Prism.

Platelet activation under arterial flow conditions culminates in the formation of thrombus at the damaged blood vessels and thereby reducing the blood supply to target tissues. Here, the effects of chrysin and Ru-thio-chrysin on thrombus formation under arterial flow conditions on collagen-coated surface were tested. DiOC6-labelled whole human blood treated with vehicle control or 100 μM chrysin or Ru-thio-chrysin was perfused over collagen-coated surfaces of Vena8 biochips and thrombus formation was monitored for 10 minutes under arterial flow conditions (20 dynes/cm^2^). The control sample showed notable level of thrombus formation over a 10 minutes period (Fig. 7A). Chrysin inhibited the thrombus growth and volume by around 50% compared to the control (Fig. 7B and C). Notably, Ru-thio-chrysin at a concentration of 100 μM significantly reduced the number of thrombi, rate of thrombus formation and volume with approximately 75% inhibition achieved at this concentration. These data corroborate the effects of Ru-thio-chrysin in whole blood (in the presence of plasma proteins and other blood cells), whereas chrysin did not exhibit similar effects.

### Chrysin and Ru-thio-chrysin affect haemostasis in mice

To determine the effects of Ru-thio-chrysin in comparison to native chrysin in the modulation of haemostasis under physiological conditions in mice, a tail-bleeding assay was performed as described previously^16^. Mice were anaesthetised prior to infusing the vehicle control or chrysin or Ru-thio-chrysin (final concentration of 25 μM) through femoral arteries. Following five minutes incubation, 1 mm of tail tip was dissected and the bleeding time was monitored. Chrysin-infused mice displayed extended bleeding time (average of 901 seconds) compared to the control group (average of 445 seconds) (Fig. 7D). Ru-thio-chrysin extended the bleeding time in mice to an average of 1096 seconds, although these effects do not significantly differ from chrysin. These data illustrate that Ru-thio-chrysin and chrysin affect haemostasis with similar efficacy in mice.

### Ru-thio-chrysin possesses enhanced bioavailability

Similar to other natural flavonoids, chrysin displayed prominent inhibitory effects in isolated platelets but their inhibitory effects were reduced under physiological conditions when PRP or whole blood were used. We hypothesised that the inhibitory effects of chrysin were reduced due to greater binding to plasma proteins such as albumin and hence, poor bioavailability, and reduced lipophilicity in order to cross cell membranes. To determine the binding ability of chrysin and Ru-thio-chrysin to human serum albumin (HSA), the HSA binding assay was performed using TRANSIL^XL^ HSA binding kit according to the manufacturer’s instructions. The binding affinities (Kd) of chrysin and Ru-thio-chrysin were determined to be 1.93 μM and 1.40 mM, respectively. Moreover, the fraction bound to plasma (fb) was found to be 99.7% for chrysin and <29.6% for Ru-thio-chrysin. These data confirm that chrysin possesses greater binding affinity towards plasma proteins, primarily albumin, than Ru-thio-chrysin.

To determine the level of uptake of chrysin and Ru-thio-chrysin in platelets, mass spectrometry-based analysis was performed. Washed human platelets were treated with chrysin or Ru-thio-chrysin (100 μM) for 5 minutes and the unbound compounds were washed prior to the quantification of the amount of chrysin and Ru-thio-chrysin present in platelets by LC-MS (Orbitrap, C8 column, Solvent system: 0.1% formic acid in water and 0.1% formic acid in acetonitrile). The uptake of Ru-thio-chrysin in platelets was found to be at 8.38 ± 1.07 μM whereas chrysin was found to be at 6.86 ± 0.27 μM as determined using standard curves (Figure S6). This indicates that Ru-thio-chrysin, which possesses higher hydrophobicity (CLogP = 7.94 as predicted using Chemdraw 16.0 software) than chrysin (CLogP = 3.04), has greater cellular permeability in platelets. These data confirm that the increased inhibitory effects observed for Ru-thio-chrysin are, indeed, due to its reduced binding to plasma proteins, greater bioavailability, and enhanced cell permeability when compared to chrysin.

### Chrysin and its synthetic derivatives display no cytotoxic effects in platelets

In order to examine whether the native chrysin and its synthetic derivatives exert any cytotoxic effects in platelets at the concentrations used in this study, the lactate dehydrogenase (LDH) assay was performed using PRP. While the positive control displayed maximal effects in platelets, chrysin and its synthetic derivatives did not show significant cytotoxic effects in platelets at the concentrations used in this study (i.e. between 6.25 and 100 μM) (Figure S7). These data confirm that the inhibitory effects of chrysin and its synthetic derivatives presented in this study were not due to the cytotoxic effects of these molecules.

## Discussion

In this study, the effects of chrysin and its chemical derivatives on the modulation of platelet function, *in vitro* thrombus formation and haemostasis were determined. Similar to a number of other dietary flavonoids such as quercetin^42–44^, tangeretin^16^, nobiletin^17^, luteolin and apigenin^18^, chrysin also exhibited inhibitory effects in various platelet functions. Chrysin affected CRP-XL-induced platelet aggregation, inside-out signalling to integrin αIIbβ3, granule secretion and integrin αIIbβ3 mediated outside-in signalling in platelets. Notably, chrysin reduced thrombus formation *in vitro* in human blood under arterial flow conditions and extended bleeding time in mice. Recently, Liu *et al*. (2016)^33^ reported the anti-platelet properties of chrysin and its possible molecular targets in platelets. Chrysin has concentration-dependently inhibited platelet activation induced by various platelet agonists including collagen, thrombin, U46619 and ADP. In addition, chrysin was found to inhibit the phosphorylation of numerous signalling proteins such as Syk, PLCγ2, AKT, PKC, ERK1/2, FAK, GSK3β and FCγRIIa consistent with the ability of flavonoids to inhibit kinase signalling. Together the previous study concluded that chrysin is involved in the modulation of platelet function by inhibiting inside-out signalling to integrin αIIbβ3 and outside-in signalling driven by the same integrin molecule^33^. Similarly, in the present study, chrysin was found to affect CRP-XL-induced inside-out signalling to integrin αIIbβ3 and phosphorylation of selective signalling molecules such as AKT, FAK and Src. In addition, chrysin was previously reported to affect platelet function *via* inhibition of cyclooxygenase activity and reduction of cAMP levels possibly by inhibiting adenylate cyclase^45^. These results indicate that chrysin is likely to have several molecular targets in platelets and thereby it modulates diverse functions of platelets enabling it to control thrombosis and haemostasis.

In general, dietary flavonoids have been shown to affect platelet function by acting as pro-oxidants in order to induce the production of nitric oxide (NO) (a potent inhibitor of platelet function through the elevation of cGMP)^46^, antioxidants by inhibiting reactive oxygen species (ROS) production, binding to cell surface receptors and affecting the integrity of the plasma membrane^18, 47^. Notably, a number of flavonoids have been shown to directly act as powerful inhibitors of numerous kinases (primarily tyrosine kinases) involved in diverse signalling pathways in platelets^18, 48^. In addition, flavonoids such as apigenin, genistein, luteolin and quercetin have also been shown to inhibit TXA_2_ receptor on platelet surface and affect its signalling. Therefore, by using a range of cellular targets, dietary flavonoids enrich the anti-platelet properties. Hence, flavonoids act as templates for the design and synthesis of therapeutically valuable compounds with specific cellular targets, and they provide a basis to determine the molecular relationships between numerous cell surface receptors, intracellular signalling proteins and dietary components^18^.

Interestingly, several studies have highlighted the potential hindrances of using dietary flavonoids as therapeutically valuable compounds for the treatment and prevention of diseases. Some of these include poor bioavailability, enhanced binding to plasma proteins and decreased hydrophobicity/ lipophilicity to cross the cell membranes^22, 36^. Therefore, several researchers have attempted to chemically modify the natural flavonoids in order to overcome these issues^23, 49^. The previous studies published on the anti-platelet effects of chrysin have mainly used washed platelets to evaluate the functions of chrysin in platelets^33, 45^. In the present study, although chrysin inhibited platelet function significantly on isolated platelets, a substantial reduction in the effects of chrysin in platelets was observed when PRP and whole blood were used in experiments. These data indicate the potential binding of chrysin to plasma proteins in PRP and also other blood cells such as leukocytes and red blood cells in whole blood, and internalisation in these cell types. The addition of sulfur groups to flavonoids has previously been shown to improve their hydrophobicity and biological effects^23^. Therefore, the present study was initiated in order to synthesise chemical derivatives of chrysin with enhanced anti-platelet effects by reducing their binding to plasma proteins and increasing their bio-availability. Initially, thio-chrysin was synthesised by replacing an oxygen molecule with a sulfur group in the basic structure of chrysin. A parallel comparison of thio-chrysin with native chrysin revealed no significant differences in their inhibitory effects when washed platelets or PRP were used in platelet aggregation assays.

In recent years, organometallic complexes have been widely considered as valuable compounds for a number of pathological conditions including cancer^27^. Indeed, platinum-based chemical agents such as cisplatin, carboplatin and oxaliplatin are being used as effective drugs in the treatment of solid tumours e.g. for testicular and other cancer types^28, 50^. Due to increased associated toxicity, lack of selectivity and side effects such as nerve damage, nausea and hair loss of platinum-based drugs, a focus on other transition metals such as ruthenium, titanium, rhodium and iridium has been initiated^24, 30, 50–53^. In order to understand the roles of ruthenium-based flavonoid complexes in the modulation of platelet function, for the first time, in this study we synthesised Ru-chrysin and Ru-thio-chrysin complexes and determined their effects on the modulation of platelet function. Interestingly, both complexes showed enhanced inhibitory effects in platelets in comparison to chrysin and thio-chrysin under physiological conditions such as in PRP and whole blood, although they exerted slightly reduced effects in washed platelets. A comparison of chrysin and Ru-thio-chrysin in thrombus formation under arterial flow conditions using whole blood has revealed the significance of ruthenium-based complexes in the modulation of platelet function under physiological conditions. Ru-thio-chrysin substantially reduced thrombus growth and volume, whilst chrysin at the same concentration only exhibited moderate reduction in thrombus volume. Notably, Ru-thio-chrysin showed similar effects in the modulation of haemostasis in mice compared to native chrysin. It appears that the combination of ruthenium with thio-chrysin exerts 10–25% greater effects compared to Ru-chrysin complex.

Since ruthenium is able to achieve several oxidation states (II, III and IV) at low energy levels under physiological conditions and interact with a range of biomolecules, it may facilitate efficient interaction of the small molecule present in the complex with its molecular targets, including targets that would normally be inaccessible for small molecules on their own^24, 50^. In addition, since ruthenium belongs to the same group within the periodic table as iron, ruthenium is known to mimic the nature of iron molecules, and therefore it has been shown to bind transferrin and thereby enhance the bioavailability of small molecules to a greater extent^54–57^. Furthermore, some ruthenium-based organometallic complexes have displayed enhanced stability in water and air^50^, which may be beneficial to exert prolonged effects of the target small molecule including chrysin. While the small molecules present in the ruthenium complexes bind to the target site, ruthenium may enhance its target-specific activities. Overall, ruthenium tends to have less severe side effects but enhanced biological properties compared to platinum-based drugs^50^. In line with these observations, our results demonstrate that Ru-thio-chrysin exhibits reduced binding ability to plasma proteins, and increased cell permeability in comparison to native chrysin. In addition, Ru-thio-chrysin shares similar molecular targets to chrysin in platelets indicating that the mechanisms through which they inhibit platelet function are unlikely to be changed. Interestingly, Ru-thio-chrysin displayed similar effects to chrysin in the modulation of haemostasis in mice and there were no toxic effects observed in platelets with the concentrations of Ru-complexes used in this study.

Together with the numerous advantages of organometallic complexes, the results of this study demonstrate the importance of using ruthenium-based organometallic complexes in the development of novel anti-platelet agents for the prevention and treatment of thrombotic diseases. Preliminary analysis of signalling cascades in this study suggests that chrysin and Ru-thio-chrysin are likely to have similar targets in platelets. However, further studies are underway to determine the specific targets of chrysin and its ruthenium-complexes in platelets. In addition, the stability of the complexes in biological systems, their circulating plasma concentrations upon oral uptake and the nature of metabolites they produce in platelets must be determined.

## Methods

### Synthesis of ruthenium-chrysin derivatives (Ru-chrysin and Ru-thio-chrysin)

- Synthesis of chlorido [(5-oxo-κO)-7-hydroxy-(2-phenyl)-4H-chromen-4-onato-κO] (η6-p-cymene)ruthenium(II) (Ru-chrysin)
  To a solution of chrysin (0.10 g, 0.39 mmol) and NaOMe (0.02 g, 0.41 mmol, 1.05 eq.) in anhydrous methanol (10 mL), [Ru(η^6^-*p*-cymene)Cl_2_]_2_ (0.13 g, 0.21 mmol, 0.55 eq.) in anhydrous dichloromethane (10 mL) was added under argon atmosphere. The reaction mixture was heated at reflux for 18 h and then the solvent was evaporated *in vacuo*. The obtained residue was dissolved in warm chloroform:methanol [9.0:1.0 v/v] (15 mL) and the solution was filtered to remove NaCl (by product) and other insoluble impurities. The solution was then concentrated (2–3 mL) and the compound was precipitated by the addition of EtOAc. The precipitated compound was filtered and recrystallized from EtOAc:CHCl_3_ (9:1 v/v) to obtain the pure product (Ru-chrysin) as an orange red solid (0.15 g, 75%). The obtained compound was suitable for X-ray diffraction studies.
**m**.**p:** 258–260 °C; ^**1**^ **H NMR:** (DMSO-d6, 400 MHz) 1.28, 1.30 (6H, 2 × s, 2 × ${{\rm{C}}{\rm{H}}}_{3{\rm{c}}{\rm{y}}{\rm{m}}}$), 2.16 (3H, s, CH_3, cym_), 2.89–2.91 (1H, m, ${{\rm{C}}{\rm{H}}}_{{\rm{c}}{\rm{y}}{\rm{m}}}$), 5.37 (2H, d, *J* *=* 8.0 Hz, H-2_cym_, H-3_cym_), 5.65 (2H, d, *J* *=* 8.0 Hz, H-5_cym_, H-6_cym_), 5.99 (1H, s, H-6), 6.05 (1H, s, H-8), 6.94 (1H, s, H-3), 7.54–7.58 (3H, m, H-3′,4′,5′), 8.01 (2H, d, *J* = 8.0 Hz, H-2′,6′), 10.29 (1H, s, OH); ^**13**^ **C NMR:** (DMSO-d6, 100 MHz) δ 17.55 (${{\rm{C}}{\rm{H}}}_{3{\rm{c}}{\rm{y}}{\rm{m}}}$), 22.02 (2 × ${{\rm{C}}{\rm{H}}}_{3{\rm{c}}{\rm{y}}{\rm{m}}}$), 30.44 (CH_cym_), 77.88 (C3), 82.54 (C8), 86.30 (C10), 90.06 (C9), 96.68 (C6), 98.12 (C3_cym_, C5_cym_), 102.41 (C2_cym_), 104.02 (C1’), 105.81 (C6_cym_), 126.22 (C3′, C5′), 129.27 (C2′, C6′, C4′), 130.52 (C1_cym_), 131.95 (C4_cym_), 158.09 (C2), 159.71 (C5), 167.76 (C7), 177.07 (**C** = O); ***m/z*** (**FTMS** + **ESI**)**:** Observed as M-Cl (C_25_H_23_O_4_Ru) requires 489.0634, found 489.0645. **Elemental analysis:** C_25_H_23_O_4_ClRu, Calculated: C-57.31%, H-4.42%, Cl-6.77%, Ru-19.29%; found: C-57.44%, H-4.25%, Cl-6.74%, Ru-19.31%. **X-ray crystal structure obtained**.
- Synthesis of chlorido [(5-oxo-κO)-7-hydroxy-(2-phenyl)-4H-chromen-4-thionato-κS] (η6-p-cymene)ruthenium(II) (Ru-thio-chrysin)

To a solution of thio-chrysin (0.10 g, 0.37 mmol) and NaOMe (0.02 g, 0.39 mmol, 1.05 eq.) in anhydrous methanol (10 mL), [Ru(η^6^-*p*-cymene)Cl_2_]_2_ (0.20 g, 0.33 mmol, 0.90 eq.) in anhydrous dichloromethane (10 mL) was added under argon atmosphere. The reaction mixture was heated at reflux for 18 h and then the solvent was evaporated *in vacuo*. The obtained residue was dissolved in warm chloroform:methanol [9.5:0.5 v/v] (15 mL) and the solution was filtered to remove NaCl (by product) and other insoluble impurities. The solution was then concentrated (2–3 mL) and the compound was precipitated by the addition of EtOAc. The precipitated compound was filtered and recrystallized from EtOAc:CHCl_3_ (9:1 v/v) to obtain the pure product (Ru-thio-chrysin) as a reddish brown solid (0.13 g, 65%). The obtained compound was suitable for X-ray diffraction studies.

**m**.**p:** decomposes at 270 °C; ^**1**^ **H NMR:** (DMSO-d6, 400 MHz) 1.21, 1.23 (6H, 2 × s, 2 × CH_3, cym_), 2.07 (3H, s, ${{\rm{C}}{\rm{H}}}_{3{\rm{c}}{\rm{y}}{\rm{m}}}$), 2.71–2.74 (1H, m, CH_, cym_), 5.31–5.68 (2H, H-2_cym_, H-3_cym_), 6.21, 7.41 (2H, H-5_cym_, H-6_cym_), 6.36 (1H, s, H-6), 6.39 (1H, s, H-8), 7.52–7.64 (3H, m, H-3′,4′,5′), 7.89 (1H, s, H-3), 8.14, 8.16 (2H, d, *J* *=* 8.0 Hz, H-2′,6′), 10.47 (1H, s, OH); ^**13**^ **C NMR:** Couldn’t be obtained. ***m/z*** (**FTMS** + **ESI**)**:** Observed as M-Cl (C_25_H_23_O_3_RuS) requires 505.0406, found 505.0407. **Elemental analysis:** C_25_H_23_O_3_ClRuS, Calculated: C-55.60%, H-4.29%, Cl-6.58%, Ru-18.72%; S-5.94% found: C-55.73%, H-4.16%, Cl-6.56%, Ru-18.71%, S-6.00%. **X-ray crystal structure obtained**.

### X-ray diffraction analysis and structure determination for Ru-chrysin and Ru-thio-chrysin

Powder X-ray diffraction data for Ru-chrysin and Ru-thio-chrysin were collected on a Bruker D8 Advance (Cu Kα1, λ = 1.54056 Å) diffractometer operating in capillary transmission mode. The diffractometer was equipped with a LynxEye detector. Monochromatic Cu Kα1 is achieved with the use of a curved Johansson type primary monochromator. Furthermore, an 8 mm detector aperture slit and a metal knife edge collimator were used to minimise air scattering.

Both samples were lightly ground prior to packing into a 0.5 mm borosilicate capillary, and the data collection was carried out at room temperature (ca. 293 K). The powder diffraction data were indexed with DICVOL91^58^ and solved using the simulated annealing (SA) approach implemented in DASH 3.3.2^34^. Previously solved crystal structures were used to derive the starting models used in the SA optimisation; the CSD reference code BENZAX^59^ was used for the Ru-coordinated cymene moiety whilst the dihydroxyflavone ligand was derived from the CSD reference code RAMGOB01^60^. Both crystal structures were subsequently refined with TOPAS (Bruker, Germany). Further crystallographic information and CIF files are given in Table S2 and Table S3, respectively. The final Rietveld fits to the powder diffraction data are shown in Figure S3.

### Human platelet preparation, aggregation assays and immunoblotting

The preparation of human platelets and aggregation assays were performed using standard protocols as described by us previously^15, 16^. Briefly, human blood was collected in 3.2% citrate containing vacutainers via venepuncture from healthy, aspirin-free individuals with informed consent in accordance with the methods approved by the University of Reading Research Ethics Committee. All methods were performed in accordance with the relevant institutional and national guidelines and regulations. Blood samples were centrifuged at 102 g for 20 minutes at room temperature to separate the PRP, which was used in aggregation, flow cytometry, static platelet adhesion, calcium mobilisation and clot retraction assays. For the preparation of washed platelets, 50 mL of blood samples were mixed with 7.5 mL of ACD (acid citrate dextrose) (20 g/L glucose, 25 g/L sodium citrate and 15 g/L citric acid) and centrifuged at 102 g for 20 minutes at room temperature. The PRP was carefully aspirated from the other blood cells prior to mixing with 3 mL of ACD and centrifuging at 1413 g for 10 minutes at room temperature. The resulting platelet pellet was re-suspended in modified Tyrodes-HEPES buffer (2.9 mM KCl, 134 mM NaCl, 0.34 mM Na_2_HPO_4_.12H_2_0, 1 mM MgCl_2_, 12 mM NaHCO_3_, 20 mM HEPES, pH 7.3) and washed by centrifuging again at 1413 g for 10 minutes. The resulting platelet pellet was suspended in modified Tyrodes-HEPES buffer at a density of 4 × 10^8^ cells/mL for aggregation, dense granule secretion, cellular uptake studies and calcium mobilisation assays. Human platelet aggregation was performed using CRP-XL as an agonist in the presence and absence of various concentrations of chrysin and its synthetic derivatives by optical aggregometry. A vehicle control [DMSO at a concentration of less than 0.1% (v/v)] was included in all the experiments. Dense granule secretion in platelets was determined by measuring the ATP release using the luciferin-luciferase reagent by lumi-aggregometry (Chrono-Log, USA). SDS-PAGE and immunoblotting analysis were performed using standard protocols^16^. The rabbit anti-phospho-specific antibodies for human AKT pS473, FAK pY397 and Src pY527 were obtained from Abcam, UK and rabbit anti-human 14-3-3ζ (Santa Cruz Biotechnology, USA) was used to detect protein 14-3-3ζ as a loading control in immunoblot assays. The Cy5-conjugated goat anti-rabbit IgG (Life technologies, UK) was used as the secondary antibody.

### Flow cytometry based assays

Fibrinogen binding (a marker for platelet inside-out signalling to integrin αIIbβ3) and P-selectin exposure (a marker for α-granule secretion) were measured by flow cytometry (Accuri C6, BD Bioscences, UK). The platelets (PRP) were treated with a vehicle control [0.1% (v/v) DMSO] or with different concentrations of chrysin and its synthetic derivatives prior to activation with CRP-XL (0.5 µg/mL). The levels of fibrinogen binding and P-selectin exposure were measured using FITC-labelled anti-human fibrinogen antibodies (Dako, UK) and PECy5-labelled CD62P antibodies (BD Biosciences, UK), respectively. The median fluorescence intensity was used to assess the levels of fibrinogen binding and P-selectin exposure on the platelet surface. The level of fluorescence obtained with the vehicle control was taken as 100% when compared with the treated samples.

### Intracellular calcium mobilisation

The intracellular calcium levels in platelets were measured using Fluo-4 AM calcium-sensitive dye by spectrofluorimetry. The PRP or washed platelets pre-incubated with Fluo-4 AM were treated with a vehicle control [0.1% (v/v) DMSO] or appropriate concentrations of chrysin or Ru-thio-chrysin prior to activating with 0.5 µg/mL CRP-XL and measuring the fluorescence continuously for 3 minutes using an excitation wavelength of 485 nm and emission at 510 nm by a Fluostar Optima spectrofluorimeter (BMG Labtech, Germany). Data were analysed by calculating the maximum level of calcium released in each sample.

### Clot retraction

PRP (200 µL) was mixed with 5 µL of red blood cells and the final volume was made to 950 µL with modified Tyrodes-HEPES buffer in the presence and absence of various concentrations of chrysin or its synthetic derivatives. Clot formation was initiated by adding 1 U/mL (50 µL) thrombin. A glass capillary was placed in middle of the tube and the clot retraction was observed over a period of 3 hours at room temperature. Clot weight was measured as a marker for clot retraction after 3 hours.

### *In vitro* thrombus formation

Human citrated blood [labelled with DiOC_6_ (Sigma Aldrich, UK)] was incubated with vehicle [0.1% (v/v) DMSO] or 100 µM of chrysin or Ru-thio-chrysin for 5 minutes and perfused over collagen coated Vena8 BioChips (Cellix Ltd, Ireland) at a shear rate of 20 dynes/cm^2^. Z-stack images of thrombi were obtained every 30 seconds for up to 10 minutes using a Nikon eclipse (TE2000-U) microscope (Nikon Instruments, UK). The median fluorescence intensity and thrombus volume were calculated by analysing the images using ImageJ.

### Tail bleeding assay

The University of Reading Local Ethical Review Panel and the British Home Office approved the tail-bleeding assay performed in this study. All methods were performed in accordance with the relevant guidelines and regulations. In brief, C57BL/6 mice (9 weeks old; Envigo, UK) were anaesthetized using ketamine (80 mg kg^−1^) and xylazine (5 mg kg^−1^) administered via intraperitoneal route 20 minutes prior to the experiment and placed on a heated pad (37 °C). The vehicle control [0.1% (v/v) DMSO] or 25 µM Chrysin (C) or Ru-thio-chrysin (Ru-tc) was injected via femoral artery 5 minutes prior to the dissection of 1 mm of tail tip using a scalpel blade. The tail tip was placed in sterile saline at 37 °C and the time to cessation of bleeding was measured up to 20 minutes.

### Platelet uptake of chrysin and Ru-thio-chrysin

Washed human platelets were treated with chrysin or Ru-thio-chrysin (100 µM) for 5 minutes and the platelets were then washed twice with modified Tyrodes-HEPES buffer by centrifugation at 1413 g for 10 minutes to remove unbound flavonoids. The compounds taken up by the platelets were extracted with methanol (400 µL). The methanol extract was dried using vacuum centrifugation and reconstituted in 200 µL of methanol for mass spectrometry (LC-MS) analysis. The concentration of chrysin and Ru-thio-chrysin in platelets were extrapolated from the standard curves of chrysin and Ru-thio-chrysin, respectively (Figure S6).

### HSA binding assay

HSA binding assay was performed using TRANSIL^XL^ binding kit (Sovicell, Germany) according to the manufacturer’s protocol. Briefly, 15 µL of 16x concentration of chrysin or Ru-thio-chrysin was added (to obtain a final concentration of 50 µM) to a column of 8 wells of the room temperature equilibrated TRANSIL assay plate containing varying amount of HSA immobilised silica beads. The plate was then incubated on a plate shaker at 100 g for 12 minutes followed by centrifugation for 10 minutes at 750 g. The supernatant (100 µL) was analysed by LC-MS to determine the affinity of chrysin and Ru-thio-chrysin based on the concentration of the free compounds in the supernatant. The Kd values for chrysin and Ru-thio-chrysin were calculated according to the instructions and algorithms supplied by the manufacturer (Sovicell, User Guide TRANSIL PPB binding kit V2.01, 2017).

### Static platelet adhesion assay

Micro titre plates (96 well) were coated with 1 μg/100 μL/well of fibrinogen, collagen or CRP-XL followed by incubation at 4 °C for overnight. Following the removal of unbound proteins/peptide, the wells were blocked with 1% bovine serum albumin in modified Tyrodes-HEPES buffer for 1 hour. The plates were then washed three times with modified Tyrode’s-HEPES buffer prior to adding human PRP (1 × 10^8^ cells/mL, 50 μL/well) and incubated at room temperature for 1 hour. Non-adhered platelets were discarded and then the wells were washed with modified Tyrodes-HEPES buffer. Citrate lysis buffer (100 μL/well) was added and incubated for 1 hour at room temperature. Finally, 100 μL of 2 M NaOH was added to all the wells to stop the reaction and the absorbance was measured at 405 nm using a Fluostar Optima spectrofluorimeter (BMG Labtech, Germany). Experiments were performed both in the absence and presence of integrilin (4 μM) (an antagonist for integrin αIIbβ3).

### LDH cytotoxicity assay

The LDH cytotoxicity assay was performed using the LDH Cytotoxicity Assay Kit (Pierce, Thermo Fisher, UK) according to the manufacturer’s instructions. In brief, the PRP was incubated at 37 °C for 30 minutes. The vehicle [0.1% (v/v) DMSO] or different concentrations of chrysin and its synthetic derivatives were added to the PRP and incubated for 5 minutes. Following incubation, the reaction mixture (provided in the kit) was added to the PRP and incubated for 30 minutes. The reaction was then stopped using a stop solution provided in the kit. The absorbance of the mixture was measured at 490–650 nm using a Fluostar Optima spectrofluorimeter (BMG Labtech, Germany). Results provided represent duplicate absorbance measures from three separate donors.

### Statistical analysis

The data obtained in this study are represented as mean ± S.D. The statistical significance between the controls and chrysin or its derivatives-treated samples was determined using one-way ANOVA. The data obtained from tail bleeding assay were analysed using a non-parametric Mann-Whitney test. All the statistical analyses were performed using GraphPad Prism 7 software (GraphPad Software Inc., USA).

### Note

CCDC deposition number for Ru-chrysin: 1495422 and Ru-thio-chrysin: 1495423.

## Electronic supplementary material

Supplementary Information
