# Supplementary material for: Ruthenium-conjugated chrysin analogues modulate platelet activity, thrombus formation and haemostasis with enhanced efficacy
Source: Sci Rep. 2017 Jul 18;7:5738. doi: 10.1038/s41598-017-05936-3 (PMC5515887; doi:10.1038/s41598-017-05936-3)
Supplement: Supplementary file 1 — Supplementary Information [file 41598_2017_5936_MOESM1_ESM.pdf]

Supplementary information

Ruthenium-conjugated chrysin analogues modulate platelet activity, thrombus formation, and haemostasis with enhanced efficacy

Divyashree Ravishankar[1*], Maryam Salamah[1*], Alda Attina[1], Radhika Pothi[1], Thomas M. Vallance[1], Muhammad Javed[1], Harry F. Williams[1], Eman M. S. Alzahrani[1], Elena Kabova[1], Rajendran Vaiyapuri[2], Kenneth Shankland[1], Jonathan Gibbins[3], Katja Strohfeldt[1], Francesca Greco[1], Helen M.I. Osborn[1♯], Sakthivel Vaiyapuri[1♯]

[1]School of Pharmacy, University of Reading, Reading, UK

[2]School of Pharmacy, University of Reading Malaysia, Johar, Malaysia

[3]Institute for Cardiovascular and Metabolic Research, School of Biological Sciences, University of Reading, Reading, UK

[*]Authors DR and MS, and [♯]HMIO and SV contributed equally to this work.

Corresponding Authors

[♯]Helen. M. I. Osborn

Tel: +44 (0)118 378 7338

E-mail address: h.m.i.osborn@reading.ac.uk

[♯]Sakthivel Vaiyapuri

Tel: +44 (0)118 378 8015

E-mail address: s.vaiyapuri@reading.ac.uk

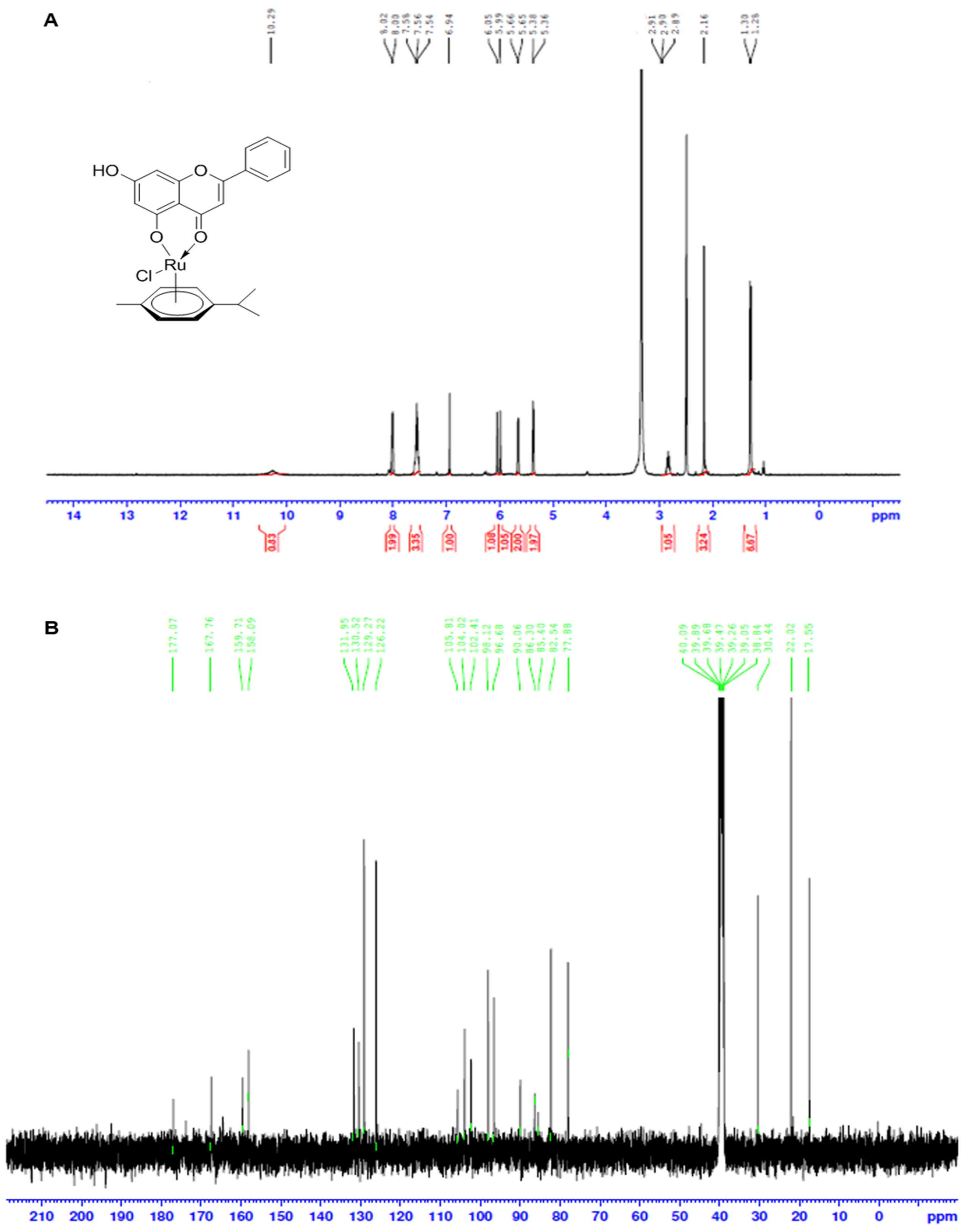

Figure S1: ^{1}H NMR (A) and ^{13}C-NMR (B) spectra of Ru-chrysin.

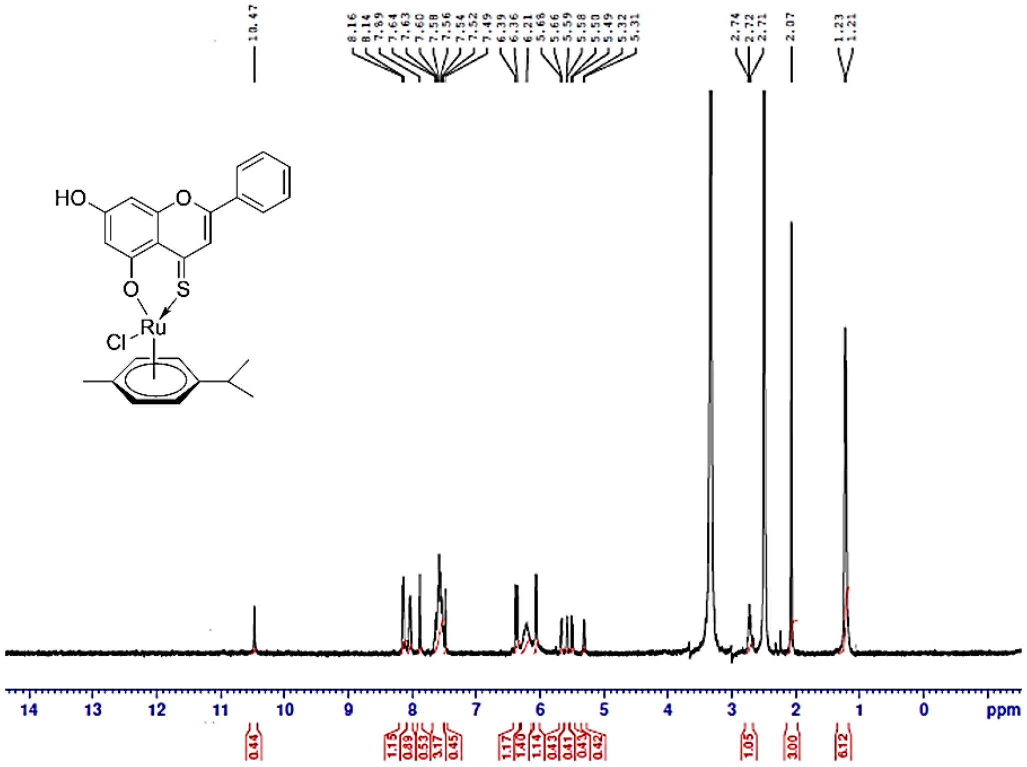

Figure S2: **^{1}H NMR spectrum of Ru-thio-chrysin.**

A

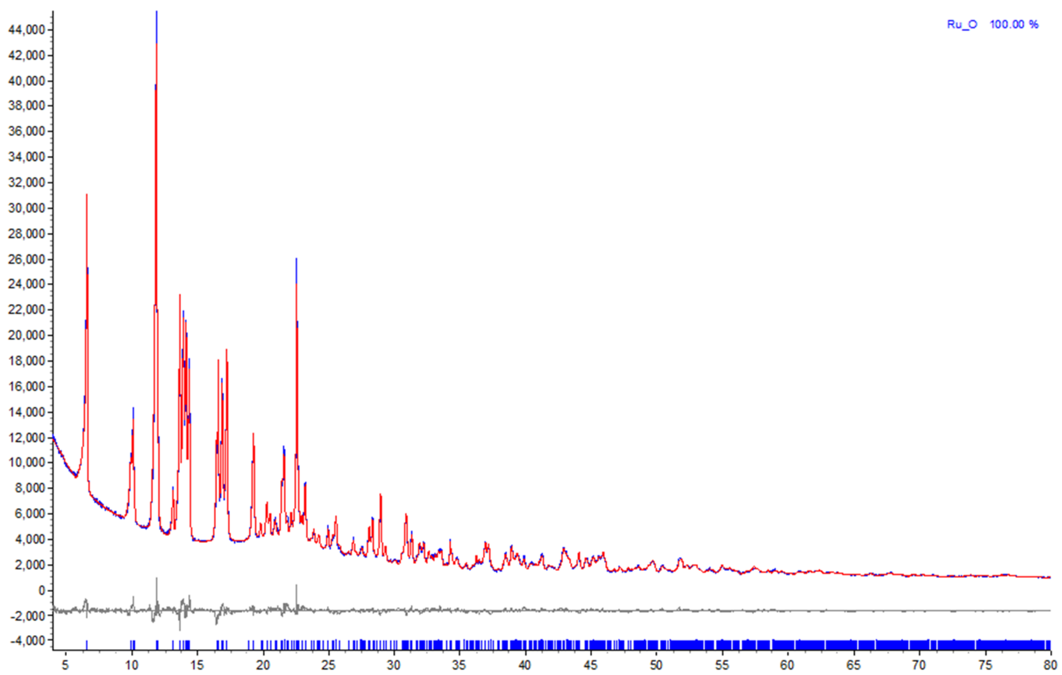

B

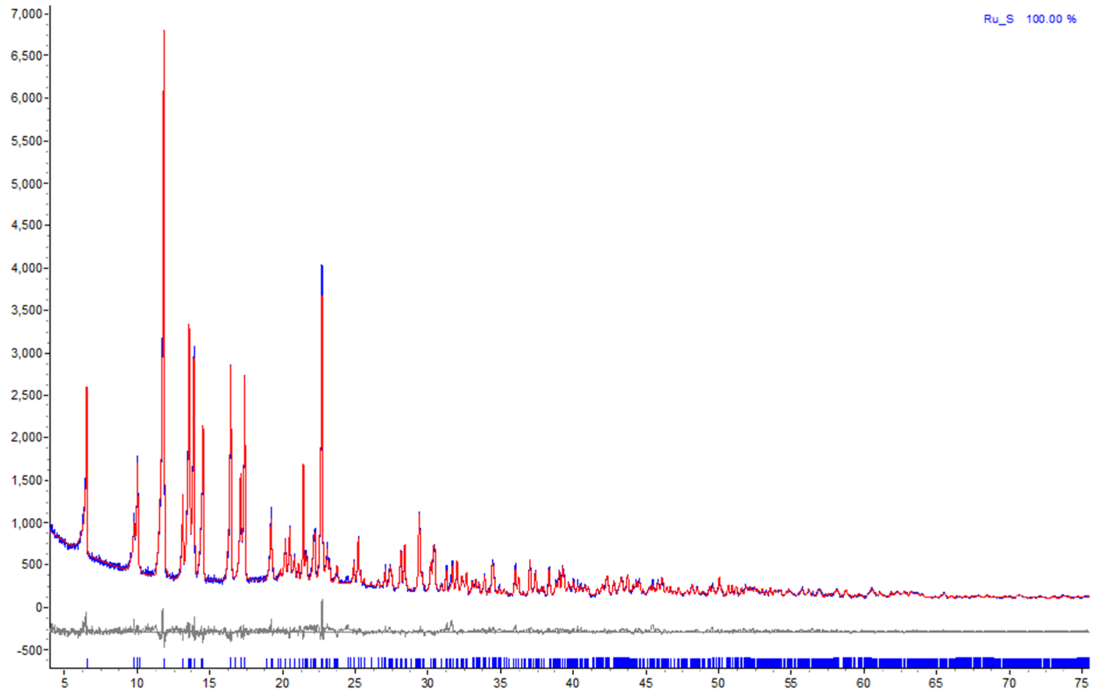

Figure S3: **Fits to the powder data for Ru-chrysin (A) and Ru-thio-chrysin (B).** The blue points are the experimental data, the red line indicates the calculated fit and the grey line indicates the difference.

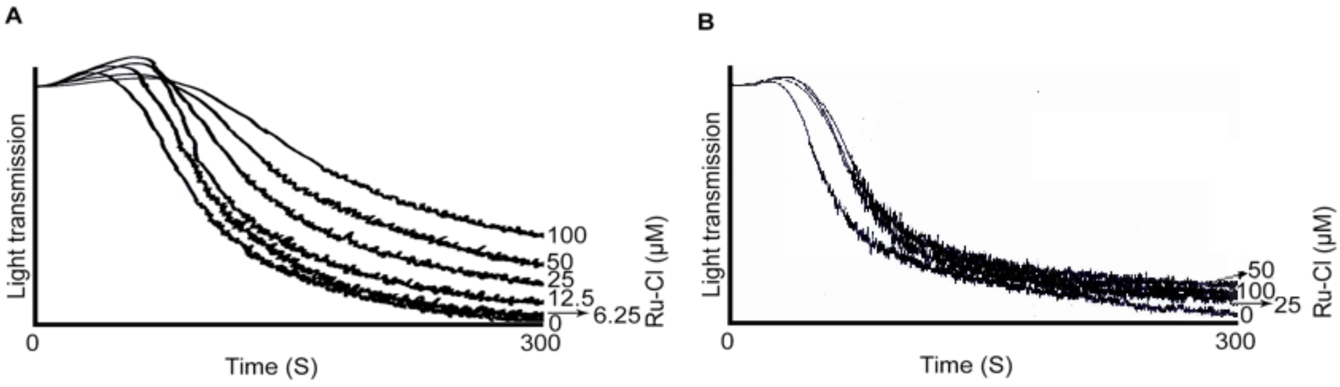

<u>Figure S4</u>: **Effects of Ru-Cl on platelet aggregation in washed platelets and PRP**. To determine if the starting material (Ru-Cl) used for the synthesis of Ru-chrysin complexes affects platelet activation, human washed platelets or PRP were treated with different concentrations of Ru-Cl for 5 minutes prior to the addition of 0.5 μg/mL CRP-XL and aggregation was monitored for 5 minutes by optical aggregometry. The traces (**A** and **B**) shown are representative of three separate experiments.

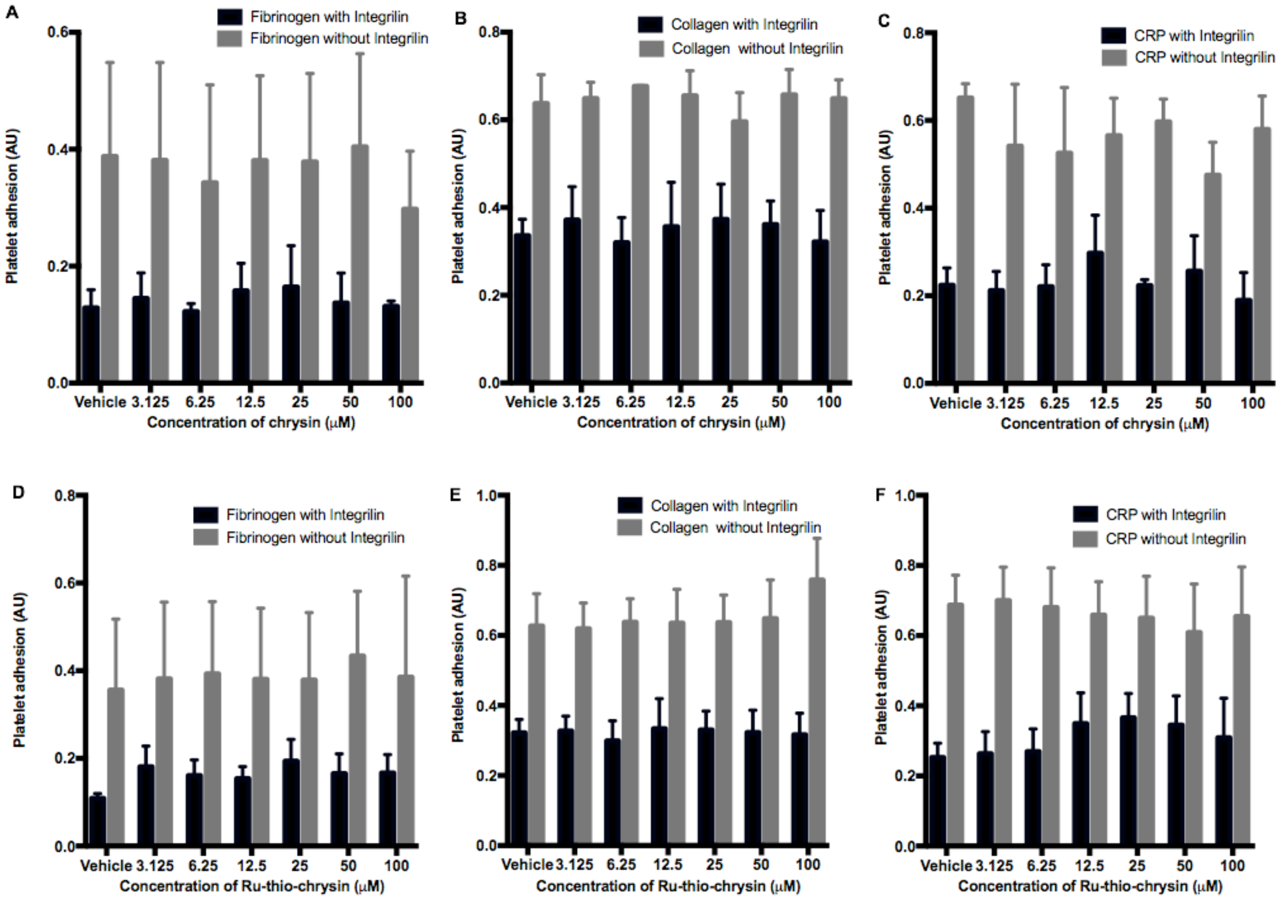

Figure S5: **Effects of chrysin and Ru-thio-chrysin on the static platelet adhesion in the presence and absence of integrilin (αIIbβ3 antagonist). (A)** Effects of chrysin on platelet adhesion to fibrinogen-coated surface, **(B)** Effects of chrysin on platelet adhesion to collagen coated-surface, **(C)** Effects of chrysin on platelet adhesion to CRP-XL-coated surface, **(D)** Effects of Ru-thio-chrysin on platelet adhesion to fibrinogen-coated surface, **(E)** Effects of Ru-thio-chrysin on platelet adhesion to collagen-coated surface, and **(F)** Effects of Ru-thio-chrysin on platelet adhesion to CRP-XL-coated surface. Data represent mean ± S.D. (n=3).

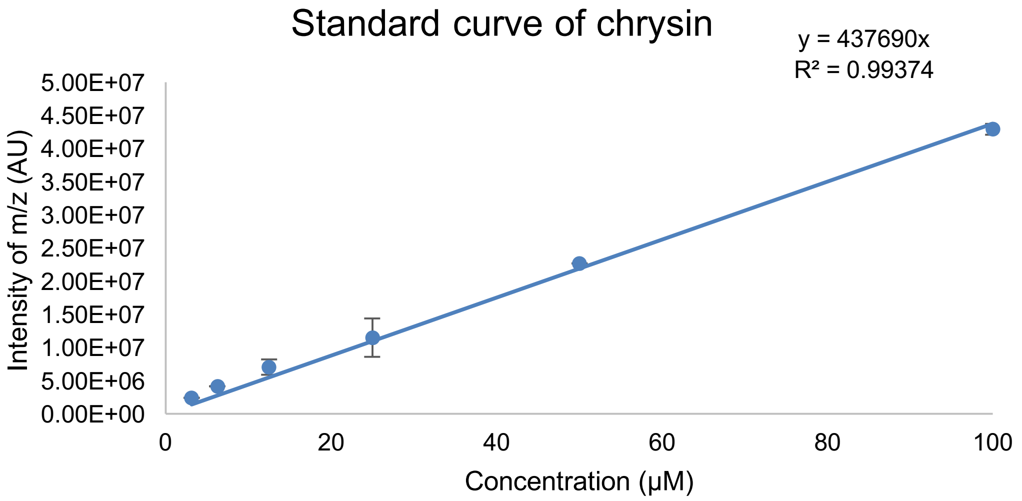

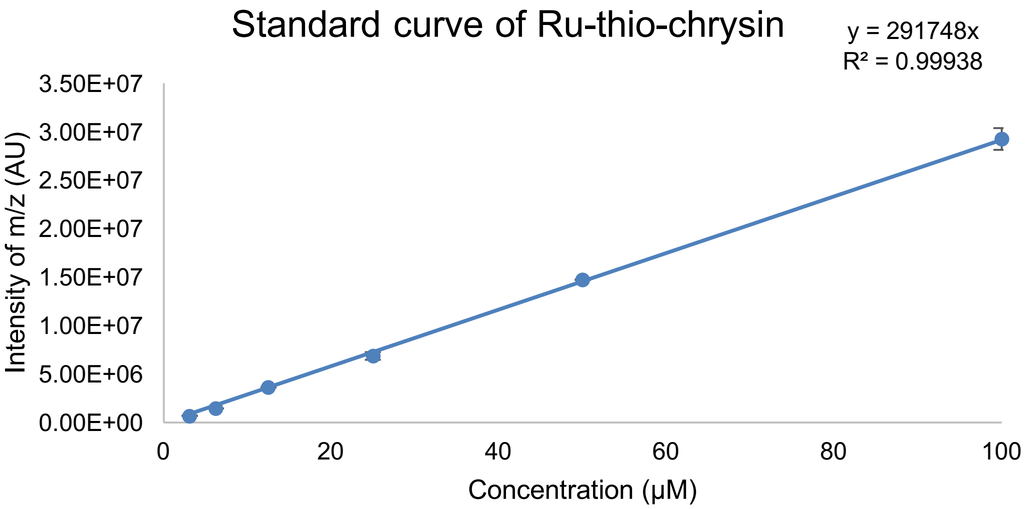

Figure S6: **Standard curves of chrysin and Ru-thio-chrysin used for the platelet uptake studies.**

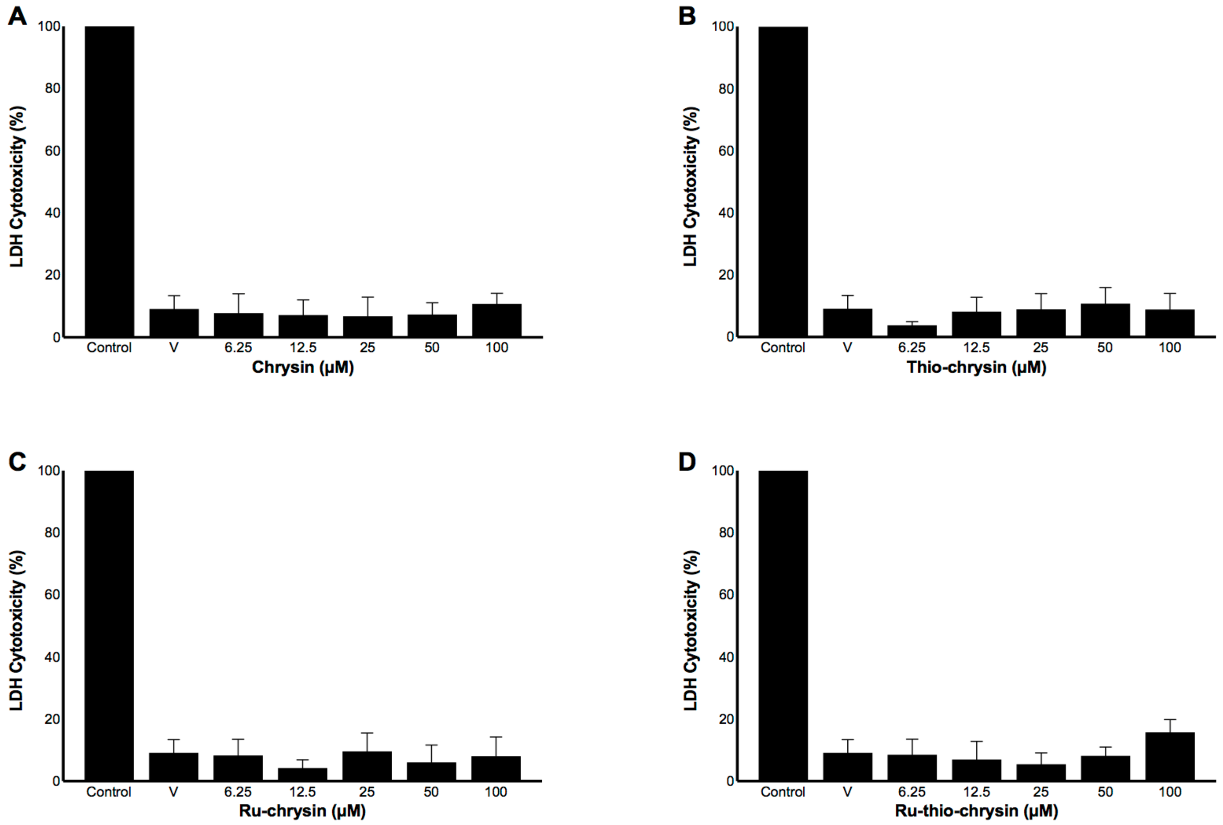

Figure S7: **Cytotoxic effects of chrysin and its synthetic derivatives in platelets.** PRP was treated with different concentrations (as used in other experiments) of chrysin or its synthetic derivatives and the LDH cytotoxicity assay was performed according to the manufacturer's instructions. The data were obtained using platelets from three separate donors and duplicate measures were used for each donor.

A

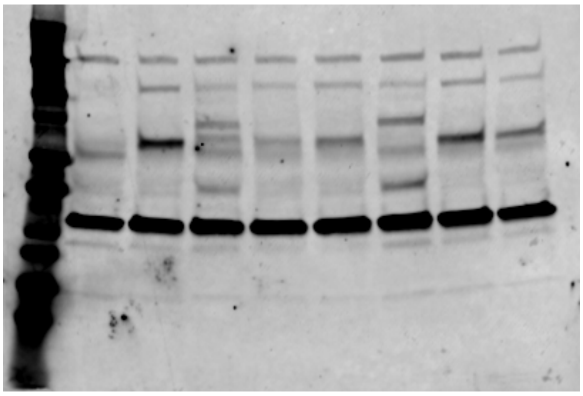

B

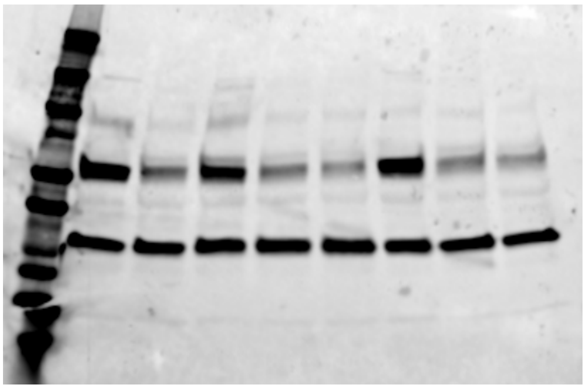

<u>Figure S8</u>: **Uncropped 'full length' images of the immunoblots presented in Figure 6C in the main article. A)** showing bands for p-FAK, p-AKT and 14-3-3ζ **and B)** showing bands for p-Src and 14-3-3ζ**.**

Table S1: **Base optimisation for the synthesis of Ru-chrysin**

Base	Number of mole equivalent	Comment
Triethyl amine	1.10	
Hunig's base	1.10	Product isolation was unsuccessful
DBU	1.10	
**NaOMe**	**1.05**	**Complete reaction conversion with 75% yield**
NaOMe	1.10	8 % uncomplexed starting material observed
NaOMe	1.25	12 % uncomplexed starting material observed
NaOMe	2.00	20% uncomplexed starting material observed

Table S2: **Summary of crystal information**

Compound	**Ru-chrysin**	**Ru-thio-chrysin**
Empirical formula	$C_{25}H_{23}ClO_4Ru$	$C_{25}H_{23}ClO_3RuS$
Formula weight (g/mol)	523.95	540.01
Crystal system	Triclinic	Triclinic
Space Group	$P\bar{1}$	$P\bar{1}$
a (Å)	8.9015(5)	9.0114(4)
b (Å)	9.1990(4)	9.2401(4)
c (Å)	14.1102(7)	14.0719(7)
α (°)	72.054(2)	72.268(3)
β (°)	89.254(2)	89.01(4)
γ (°)	89.346(4)	86.613(4)
V ($Å^3$)	1099.07(10)	1114.10(9)
Z	2	2
Z'	1	1
Wavelength (Å)	1.5406	1.5406
Data range, 2θ (°)	4-80	4-75.5
Scan step increment, 2θ (°)	0.017	0.017
No. of data points	4470	4174
No. of reflections	1355	1195
Temperature	Room temperature	Room temperature
Total collection time	20 hours	20 hours
VCT ranges, 2θ (°)	4.00 - 23.00<br>23.00 - 41.99<br>41.99 - 60.99<br>60.99 – 80.02	4.00 - 23.00<br>23.00 - 41.99<br>41.99 - 60.99<br>60.99 - 75.55
Number of parameters	43	50
R_{wp}	2.96	5.86

Table S3: **CIF files for Ru-chrysin and Ru-thio-chrysin**

**Ru-chrysin**	**Ru-thio-chrysin**
CIF file	CIF file
Empirical formula $C_{25}H_{22}ClO_4Ru$	Empirical formula $C_{25}H_{22}ClO_3RuS$
Formula weight 522.97	Formula weight 539.03
data_global	data_
_symmetry_cell_setting triclinic	_chemical_name_mineral ?Ru_S?
_symmetry_space_group_name_H-M 'P -1'	_cell_length_a 9.01142
_symmetry_Int_Tables_number 2	_cell_length_b 9.24012
loop_	_cell_length_c 14.07193
_symmetry_equiv_pos_site_id	_cell_angle_alpha 72.26756
_symmetry_equiv_pos_as_xyz	_cell_angle_beta 89.0045
1 x,y,z	_cell_angle_gamma 86.61326
2 -x,-y,-z	_cell_volume 1114.103
_cell_length_a 8.9040	_symmetry_space_group_name_H-M P-1
_cell_length_b 9.2014	loop_
_cell_length_c 14.1157	_symmetry_equiv_pos_as_xyz
_cell_angle_alpha 72.0429	'-x, -y, -z'
_cell_angle_beta 89.2407	'x, y, z'
_cell_angle_gamma 89.3621	loop_
_cell_volume 1100.03	_atom_site_label
loop_	_atom_site_type_symbol
_atom_site_label	_atom_site_symmetry_multiplicity
_atom_site_type_symbol	_atom_site_fract_x
_atom_site_fract_x	_atom_site_fract_y
_atom_site_fract_y	_atom_site_fract_z
_atom_site_fract_z	_atom_site_occupancy
O1 O 0.37939 0.98624 0.68221	_atom_site_B_iso_or_equiv
O2 O 0.13210 0.59804 0.73410	Ru1 Ru 0 0.6369923 0.3824521 0.8351686 1
O3 O 0.23062 0.50476 0.91652	2.77991
H2 H 0.41355 0.58337 1.03518	Cl1 Cl 0 0.8865657 0.3500722 0.7722879 1
O4 O 0.56018 0.83479 1.00980	2.77991
C1 C 0.20893 0.71768 0.71789	C25Z C 0 0.4574788 0.2791805 0.7782884 1
C2 C 0.21526 0.83272 0.62278	2.77991
C3 C 0.29826 0.96111 0.60846	C24Z C 0 0.4023665 0.3677133 0.8402461 1
C4 C 0.38075 0.87963 0.77464	2.77991
C5 C 0.29786 0.74483 0.79503	C26Z C 0 0.5690891 0.1648146 0.8193778 1
C6 C 0.30687 0.63714 0.89112	2.77991
C7 C 0.39900 0.66475 0.96183	C29Z C 0 0.4009655 0.3100931 0.6725288 1
C8 C 0.47563 0.80398 0.93971	2.77991
C9 C 0.46679 0.91322 0.84630	C23Z C 0 0.455444 0.3414904 0.937317 1
C10 C 0.31270 1.08471 0.51375	2.77991
C11 C 0.40406 1.21057 0.50626	H25Z H 0 0.3277823 0.4463082 0.814849 1
C12 C 0.42216 1.32365 0.41508	3.335893
C13 C 0.34780 1.31400 0.33107	H26Z H 0 0.60996 0.1050612 0.7800812 1
C14 C 0.25362 1.19086 0.33897	3.335893
C15 C 0.23632 1.07687 0.42922	C27Z C 0 0.6219293 0.1371136 0.918534 1
H1 H 0.14983 0.81635 0.56278	2.77991
H3 H 0.55409 0.75036 1.07113	H31Z H 0 0.3797043 0.4224891 0.645094 1
H4 H 0.52660 1.02026 0.82903	3.335893
H5 H 0.46635 1.21789 0.56999	C30Z C 0 0.5156391 0.265952 0.6033284 1
H6 H 0.50295 1.41281 0.41038	2.77991
H7 H 0.36249 1.40535 0.26176	C31Z C 0 0.256016 0.2352868 0.6682712 1
H8 H 0.19031 1.18010 0.27652	2.77991
H9 H 0.15566 0.98876 0.43109	C22Z C 0 0.5662211 0.2237412 0.9791072 1
C16 C 0.05301 0.22436 0.97462	2.77991
C17 C 0.11993 0.14697 0.91118	H24Z H 0 0.4169028 0.4032959 0.9759972 1
H10 H 0.19408 0.07042 0.93711	3.335893
C18 C 0.07833 0.18174 0.81127	H27Z H 0 0.6964228 0.05838097 0.9442523 1
H11 H 0.12422 0.12737 0.77117	3.335893
C19 C -0.03157 0.29701 0.76799	H32Z H 0 0.6085524 0.3149027 0.6059567 1

C20 C -0.09523 0.37518 0.83063<br>H12 H -0.16703 0.45393 0.80428<br>C21 C -0.05402 0.33862 0.93256<br>H13 H -0.09990 0.39267 0.97297<br>C22 C 0.10262 0.18984 1.08024<br>H14 H 0.05872 0.09295 1.12076<br>H15 H 0.21246 0.18189 1.08289<br>H16 H 0.06925 0.27216 1.10627<br>C23 C -0.07417 0.33563 0.65915<br>H17 H -0.07846 0.44961 0.63179<br>C24 C -0.22939 0.27690 0.64413<br>H18 H -0.30413 0.31271 0.68363<br>H19 H -0.25556 0.31552 0.57346<br>H20 H -0.22784 0.16489 0.66566<br>C25 C 0.04231 0.28132 0.59742<br>H21 H 0.00945 0.30935 0.52810<br>H22 H 0.13887 0.32968 0.60048<br>H23 H 0.05343 0.17009 0.62401<br>Ru1 Ru 0.14535 0.39004 0.83907<br>Cl1 Cl 0.39976 0.34959 0.76894<br>#END	3.335893<br>H33Z H 0 0.4775108 0.2995315 0.5348208 1 3.335893<br>H34Z H 0 0.5342749 0.1551508 0.6249391 1 3.335893<br>H35Z H 0 0.2246112 0.2590646 0.5986326 1 3.335893<br>H36Z H 0 0.1792477 0.2739494 0.705849 1 3.335893<br>H37Z H 0 0.2710306 0.1248141 0.6975876 1 3.335893<br>C28Z C 0 0.6257534 0.2007365 1.081626 1 2.77991<br>H28Z H 0 0.5533166 0.1487814 1.131437 1 3.335893<br>H29Z H 0 0.6436851 0.2997341 1.090099 1 3.335893<br>H30Z H 0 0.7193087 0.1386623 1.09051 1 3.335893<br>C3Z C 0 0.8108372 0.9856461 0.6004298 1 2.77991<br>O1Z O 0 0.8846463 0.9984647 0.6801678 1 2.77991<br>C2Z C 0 0.7282533 0.8645859 0.6059281 1 2.77991<br>C10Z C 0 0.8325035 1.113465 0.5098423 1 2.77991<br>C4Z C 0 0.8786823 0.8863789 0.7698375 1 2.77991<br>C1Z C 0 0.7146115 0.745391 0.6970068 1 2.77991<br>H1Z H 0 0.6772131 0.8590112 0.55044 1 3.335893<br>C11Z C 0 0.9239385 1.230146 0.5113093 1 2.77991<br>C15Z C 0 0.7630086 1.118901 0.4203211 1 2.77991<br>C5Z C 0 0.7957367 0.7585269 0.7812596 1 2.77991<br>C9Z C 0 0.9574634 0.9073105 0.8480204 1 2.77991<br>S1Z S 0 0.6118052 0.5937723 0.7078095 1 2.77991<br>C12Z C 0 0.948924 1.347167 0.4240151 1 2.77991<br>H6Z H 0 0.9728855 1.227527 0.5694453 1 3.335893<br>C14Z C 0 0.7871304 1.236758 0.3339795 1 2.77991<br>H10Z H 0 0.6941326 1.05009 0.4159195 1 3.335893<br>C6Z C 0 0.7970414 0.6445944 0.8749449 1 2.77991<br>C8Z C 0 0.9588493 0.7921122 0.9389063 1 2.77991<br>H5Z H 0 1.008978 0.9948207 0.8390619 1 3.335893<br>C13Z C 0 0.8813867 1.35064 0.3350227 1 2.77991<br>H7Z H 0 1.018095 1.417058 0.4259873 1 3.335893<br>H9Z H 0 0.7372335 1.236479 0.2768013 1 3.335893

	O3Z O 0 0.7213494 0.52012 0.8916043 1 2.77991<br>C7Z C 0 0.8819506 0.659407 0.9521747 1 2.77991<br>O5Z O 0 1.0363 0.8105829 1.015302 1 2.77991<br>H8Z H 0 0.898483 1.4314 0.2783113 1 3.335893<br>HZ H 0 0.8880157 0.5818785 1.012493 1 3.335893<br>H4Z H 0 1.015864 0.8967349 1.020202 1 3.335893<br>#END
